# Assembly of Dy_60_ and Dy_30_ cage-shaped nanoclusters

**DOI:** 10.1038/s42004-020-0276-3

**Published:** 2020-03-06

**Authors:** Zhi-Rong Luo, Hai-Ling Wang, Zhong-Hong Zhu, Tong Liu, Xiong-Feng Ma, Hui-Feng Wang, Hua-Hong Zou, Fu-Pei Liang

**Affiliations:** 1 State Key Laboratory for Chemistry and Molecular Engineering of Medicinal Resources, School of Chemistry and Pharmacy of Guangxi Normal University, Guilin, 541004 China; 2 Guangxi Key Laboratory of Electrochemical and Magnetochemical Functional Materials, College of Chemistry and Bioengineering, Guilin University of Technology, Guilin, 541004 China

**Keywords:** Organometallic chemistry, Self-assembly

## Abstract

Rapid kinetics, complex and diverse reaction intermediates, and difficult screening make the study of assembly mechanisms of high-nuclearity lanthanide clusters challenging. Here, we synthesize a double-cage dysprosium cluster [Dy_60_(H_2_**L**^**1**^)_24_(OAc)_71_(O)_5_(OH)_3_(H_2_O)_27_]·6H_2_O·6CH_3_OH·7CH_3_CN (**Dy**_**60**_) by using a multidentate chelate-coordinated diacylhydrazone ligand. Two **Dy**_**30**_ cages are included in the **Dy**_**60**_ structure, which are connected via an OAc^−^ moiety. The core of **Dy**_**60**_ is composed of 8 triangular Dy_3_ and 12-fold linear Dy_3_ units. We further change the alkali added in the reaction system and successfully obtain a single cage-shaped cluster [Dy_30_(H_2_**L**^**1**^)_12_(OAc)_36_(OH)_4_(H_2_O)_12_]·2OH·10H_2_O·12CH_3_OH·13CH_3_CN (**Dy**_**30**_) with a perfect spherical cavity, which could be considered an intermediate in **Dy**_**60**_ formation. Time-dependent, high-resolution electrospray ionization mass spectrometry (HRESI-MS) is used to track the formation of **Dy**_**60**_. A possible self-assembly mechanism is proposed. We track the formation of **Dy**_**30**_ and the six intermediate fragments are screened.

## Abstract (web summary)

High-nuclearity lanthanide clusters can exhibit interesting properties such as magnetism, but the mechanisms by which they assemble are poorly understood. Here, the formation of two dysprosium nanoclusters containing 30 or 60 atoms is followed over time by mass spectrometry and proposed to follow a seven-step assembly mechanism.

## Introduction

Over the past few decades, the design and synthesis of high-nuclearity lanthanide clusters with complex structures and interesting properties has been extremely active (Supplementary Table 1)^1–5^. To date, a variety of high-nuclearity lanthanide clusters (e.g., **Gd**_**140**_, **Ln**_**104**_, **Dy**_**76**_, **Dy**_**72**_, **Er**_**60**_, **Gd**_**60**_, and **Ln**_**48**_) have been designed and synthesized, and their magnetic, optical, and catalytic applications have been successfully extended^1–12^. Although considerable progress has been made in the synthesis and performance expansion of high-nuclearity lanthanide clusters, there are still only two relatively mature synthetic strategies, namely ligand-controlled hydrolysis and anionic template strategies^1,2^. In ligand-controlled hydrolysis, Ln(III) ion is initially combined with water and allowed to further participate in hydrolysis to obtain a template precursor, such as linear {Ln_2_(*μ*_2_-OH)}, triangular {Ln_3_(*μ*_3_-OH)} or {Ln_3_(*μ*_3_-OH)_2_}, tetrahedral {Ln_4_(*μ*_3_-OH)_4_}, trigonal bipyramidal {Ln_5_(*μ*_3_-OH)_6_}, square pyramidal {Ln_5_(*μ*_3_-OH)_4_(*μ*_4_-O)}, and octahedral {Ln_6_(*μ*_3_-OH)_8_(*μ*_6_-O)} precursors^1^. In 2014, Long and colleagues^7^ hydrolyzed Ln(ClO_4_)_3_ in the presence of acetate to obtain Ln-exclusive high-nuclearity cluster **Ln**_**104**_ containing 24 independent units [Ln_5_(*μ*_3_-OH)_4_(*μ*_4_-O)]^9+^. Zheng and colleagues^9^ finally synthesized the tubular dysprosium cluster **Dy**_**72**_ by controlling the hydrolysis of Dy(III) ions via *N*-methyldiethanolamine. The ligand-controlled hydrolysis process is mainly determined by the hydrolysis of Ln(III) ions^1,2^. During hydrolysis, different shapes of hydroxyl intermediate templates are generated to further aggregate and construct high-nuclearity lanthanide clusters^1^. Therefore, the synthesis of high-nuclearity lanthanide clusters via ligand-controlled hydrolysis is sensitive to the selection of reaction raw materials and reaction conditions, and different hydroxy intermediate templates are easily generated during the reaction to make the reaction disordered^1,8^. As such, tracking the reaction process and exploring its self-assembly mechanism have become difficult tasks. In the anionic template strategy, the formation of high-nuclearity lanthanide clusters is hindered by the mutual exclusion of positive charges between Ln(III) ions. Therefore, introducing a small volume of anion as a reaction building block is an effective strategy for solving the abovementioned problems^1,2^. In comparison with a single anion template, multiple anions or mixed different anions as templates can disperse the positive charge of Ln(III) ions in the cluster and are conducive to the synthesis of high-nuclearity lanthanide clusters; such anions further act as a negative charge to balance the positive charge in the cluster^1,8^. In 2015, Zhao and colleagues^13^ formed eight CO_3_^2−^ templates via in-situ ligand decomposition and obtained a cage-shaped cluster **Ln**_**60**_. Thereafter, Tong and colleagues^14^ induced the synthesis of two Gd(III) clusters **Gd**_**38**_ and **Gd**_**48**_ by using Cl^−^ and NO_3_^−^ templates. Song and colleagues^11^ used a combination of OAc^−^ and CO_3_^2^^−^ mixed templates to synthesize nanocluster **Gd**_**60**_. In 2019, Bu and colleagues^8^ used mixed anions Cl^−^ and CO_3_^2−^ as templates to induce the formation of basic construction units [Dy_3_(*μ*_3_-OH)_4_] and [Dy_5_(*μ*_4_-O)(*μ*_3_-OH)_8_], and synthesized **Dy**_**76**_. When synthesizing high-nuclearity lanthanide clusters through the anionic template strategy, the reaction is complicated due to the diversity of anion selection and the uncontrollability of the template^1,2^. Therefore, selecting the appropriate method and means to track self-assembly is difficult.

In the design and synthesis of high-nuclearity lanthanide clusters, multidentate chelating ligands can rapidly bind and stabilize Ln(III) ions, making their self-assembly regular^15–19^. The selection of suitable multidentate chelating ligands is instrumental for studies on the assembly mechanism of high-nuclearity lanthanide clusters^20,21^. The acylhydrazone ligands with strong chelating ability, multiple coordination modes, and easily changing coordination configurations have made progress in the synthesis of low-nuclear lanthanide clusters^15–17^; however, their use in the design and synthesis of giant lanthanide clusters is rare^18,19^. In 2016, we synthesized the largest lanthanide wheel-like cluster **Gd**_**18**_ at the time by using acylhydrazone ligands^18^. We used different lanthanide metal salts and obtained **Gd**_**11**_ with a high magnetocaloric effect under the same reaction conditions^19^. In the hydrothermal/solvothermal “black box” system under certain temperature and pressure conditions, the temperature and pressure in a closed system make the collision between reaction species confusing. Thus, the exploration of the assembly mechanism of coordination molecular clusters is slow^20–35^. Although complex, progress has been made in polyoxometalates^22–25,36,37^, coordination supramolecular systems^38,39^, coordination molecular cages^26,40–45^, 3*d* metal–ion clusters^30–33^, and so on^46,47^. High-valence Ln(III) ions usually have complex coordination configurations, rich and diverse coordination modes, and coordination balls that are prone to distortion^20,21,48–50^. Such drawbacks lead to the formation of several types of assembly methods in the synthesis of high-nuclearity lanthanide clusters, such as multi-template induction and stepwise assembly^20,21,48–50^. Given that various assembly methods exist, the formation process of high-nuclearity lanthanide clusters is confusing and exploring the assembly mechanism is difficult^20,21,48–50^. Therefore, studies on the assembly mechanism of lanthanide clusters are limited^20,21,48–51^. In 2018, Long and colleagues^51^ used high-resolution electrospray ionization mass spectrometry (HRESI-MS) to guide the assembly and synthesis of the wheel-like **Eu**_**24**_**Ti**_**8**_ cluster. We initially used HRESI-MS to track the assembly of Dy-exclusive coordination cluster **Dy**_**3**_ and proposed its assembly mechanism^20^. In 2019, we used HRESI-MS to track the stepwise assembly of **Dy**_**10**_ with multiple relaxation behavior^21^. Subsequently, we traced the assembly of a nanocluster **Dy**_**12**_ composed of four vertices sharing Dy_4_(*μ*-OH)_4_ via HRESI-MS. The relationship between stepwise and template assembly in the formation of high-nuclearity lanthanide clusters was described^48^. Finally, we tracked the competitive assembly process of mixed ligands and in-situ Schiff base replacement in the formation of lanthanide clusters via HRESI-MS^49,50^.

Here we synthesize the Dy-exclusive double-cage-shaped cluster **Dy**_**60**_ under solvothermal conditions by using diacylhydrazone ligands with multidentate chelating coordination (Supplementary Figs. 1 and 2). Tetrabutyl ammonium hydroxide (Bu_4_NOH) in the reaction of **Dy**_**60**_ synthesis is changed to LiOH, whereby the stable intermediate **Dy**_**30**_ is obtained under the same reaction conditions. The stoichiometric reaction of **Dy**_**30**_, Bu_4_NOH, and Dy(OAc)_3_·4H_2_O under solvothermal conditions resulted in the high-yield synthesis of the cluster **Dy**_**60**_ (Supplementary Fig. 2). Time-dependent HRESI-MS track the formation of **Dy**_**60**_. We proposed the **Dy**_**60**_ possible assembly mechanism as follows: H_6_**L**^**1**^ → Dy**L**^**1**^ → Dy_2_**L**^**1**^ → Dy_3_**L**^**1**^ → Dy_4_**L**^**1**^ → Dy_5_(**L**^**1**^)_2_ → Dy_30_(**L**^**1**^)_12_ → Dy_60_(**L**^**1**^)_24_. We used the same method to track the formation of **Dy**_**30**_ and further verify the assembly mechanism of **Dy**_**60**_. A possible assembly mechanism of **Dy**_**30**_ is as follows: H_6_**L**^**1**^→ Dy**L**^**1**^→Dy_2_**L**^**1**^→Dy_3_**L**^**1**^→Dy_4_**L**^**1**^→Dy_5_(**L**^**1**^)_2_→Dy_30_(**L**^**1**^)_12_.

## Results and discussion

### Crystal structural analysis

**Fig. 1:**
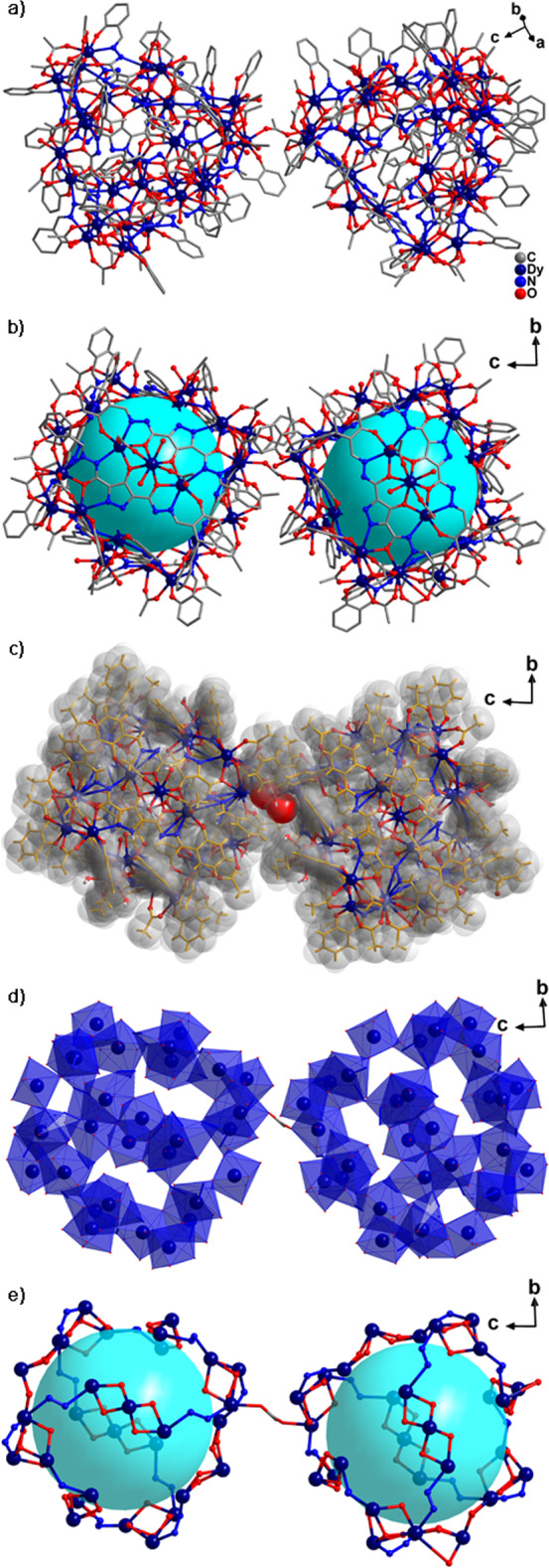
Crystal structure of Dy_60_. **a** Molecular structure of the cage-shaped **Dy**_**60**_. **b** Illustration of **Dy**_**60**_. **c** Space-filling mode of **Dy**_**60**_, which exhibited all ligands protected the two main cores. **d** Polyhedron of Dy(III) ions in Dy···Dy cage. **e** Cage mode in Dy/O/N core.

The nanocluster **Dy**_**60**_ was synthesized using Dy(OAc)_3_·4H_2_O, (*N*′^3^*E*,*N*′^5^*E*)-4-hydroxy-*N*′^3^,*N*′^5^-bis(2-hydroxybenzylidene)-1*H*-pyrazole-3,5-dicarbohydrazide (H_6_**L**^1^), and tetrabutyl ammonium hydroxide (Bu_4_NOH) in the presence of mixed solvent (CH_3_OH:CH_3_CN = 1 : 1, 1.6 mL) under solvothermal conditions for 2 days at 80 °C. Single-crystal X-ray diffraction pattern revealed that nanocluster **Dy**_**60**_ crystallized in the triclinic *P*-1 space group (Supplementary Table 2) containing 60 Dy(III) ions, 24 (H_2_**L**^1^)^4‒^ ligands, 5 *µ*_3_-O^2‒^, 3 OH^−^, one bridged *µ*_2_-OAc^−^, 70 OAc^−^, and 27 H_2_O molecules (Fig. 1a). The size of nanocluster **Dy**_**60**_ was 5.2 nm × 2.7 nm and the diameter of every cavity was 1.5 nm (Dy13···Dy21) (Fig. 1b). The volume of the above spherical cavity is 2.4 nm^3^. The periphery of the Dy/O/N core of **Dy**_**60**_ was covered by 24 (H_2_**L**^1^)^4−^ ligands forming the organic ligand structure that protected the Dy/O/N core of **Dy**_**60**_ in the periphery (Fig. 1c, d). In this nano-sized Dy(III) cluster, the main core of **Dy**_**60**_ was composed of eight triangular Dy_3_ and 12-fold linear Dy_3_ units. In the Dy/O/N core of **Dy**_**30**_, each Dy(III) ion in the four triangular lattices Dy_3_ was connected by a folding linear Dy_3_ through the ‒N-N‒ bridging on the ligand. The two cage-shaped **Dy**_**30**_ was formed **Dy**_**60**_ by bridging the Dy(III) ions of the two triangular lattices by one *µ*_2_-OAc^−^ (Fig. 1e). In the Dy/O core of **Dy**_**60**_, the bond distances of Dy···Dy in triangular lattices Dy_3_ were within the limits of 3.805–3.939 Å and the bond distances of Dy···Dy were within the limits of 3.707–3.836 Å in the folding linear Dy_3_ (Supplementary Fig. 3a). The ligand in **Dy**_**60**_ showed one coordination mode in the structure: *μ*_5_-*η*^1^:*η*^1^:*η*^2^:*η*^2^:*η*^1^:*η*^1^:*η*^2^:*η*^1^:*η*^1^ (Supplementary Fig. 3b). OAc^−^ is mainly used for short-bridged ligands during **Dy**_**60**_ formation. OAc^−^ can coordinate with Dy(III) ions to make them saturated and bridge several adjacent Dy(III) ions in the cluster, thereby ensuring the stability of the cage high-core cluster **Dy**_**60**_. A structural figure of Dy_60_ with probability ellipsoids is given in Supplementary Fig. 4.

High nuclear lanthanide clusters usually form ring-type (**Gd**_**140**_)^6^, hamburger-type^8^, disordered-type^52^, and ellipsoid configuration^53^ structures through simple anion ligands (Supplementary Table 1). However, the formation of cage-shaped clusters is rare, in which the high nuclear caged lanthanide cluster **Er**_**60**_ is a sodalite caged structure composed of 24 [Er_4_(*μ*_3_-OH)_4_]^8+^ cubanes by vertex sharing^10^. Cage-shaped dysprosium high-nuclearity clusters, such as **Dy**_**17**_, **Dy**_**24**_, **Dy**_**26**_, **Dy**_**27**_, and **Dy**_**104**_, have been reported^54–57^. **Dy**_**104**_ is composed of four single cage-shaped **Dy**_**26**_ bridged by ligands.

The nanocluster **Dy**_**60**_ can be interpreted as two **Dy**_**30**_ connected by one bridged OAc^−^, thereby providing a new structural model for the lanthanide clusters. The single cage-shaped nanocluster **Dy**_**30**_ should be synthesized to verify the rationality of connecting two nanocluster **Dy**_**30**_ with one OAc^−^ bridge. We attempted to block the formation of the final state by shortening the reaction time via the same method for the synthesis of **Dy**_**60**_. However, we did not obtain the crystal of **Dy**_**30**_ under the same experimental conditions. A single cage-shaped nanocluster **Dy**_**30**_ was successfully obtained by replacing Bu_4_NOH with LiOH during nanocluster **Dy**_**60**_ synthesis. We also successfully obtained the cluster **Dy**_**60**_ by reacting **Dy**_**30**_, Bu_4_NOH, and Dy(OAc)_3_·4H_2_O under solvothermal conditions.

**Fig. 2:**
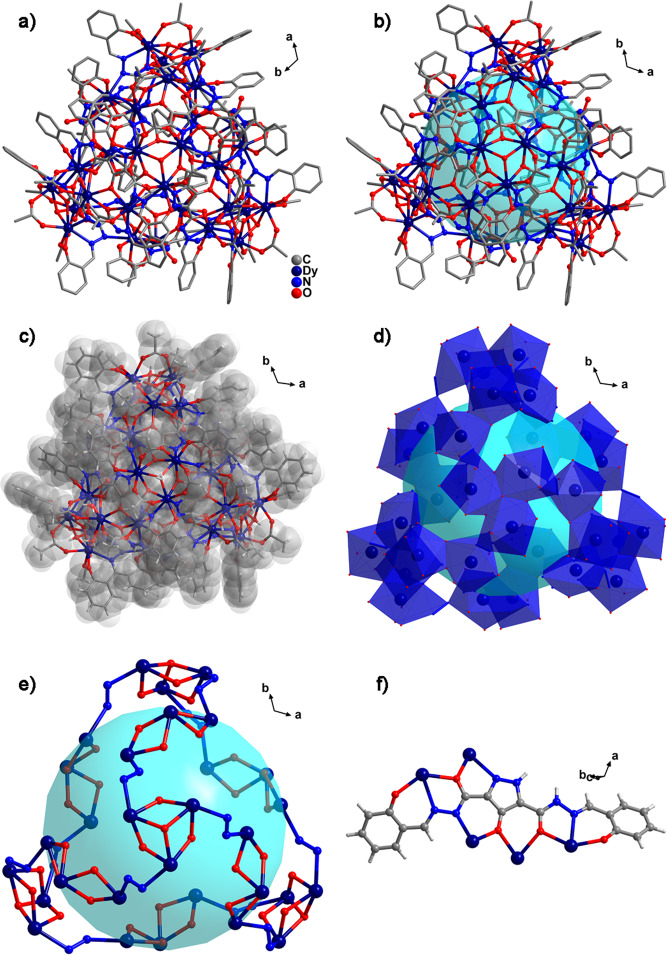
Crystal structure of Dy_30_. **a** Molecular structure of cage-shaped **Dy**_**30**_. **b** Illustration of **Dy**_**30**_. **c** Space-filling mode of **Dy**_**30**_. **d** Polyhedron of Dy(III) ions in Dy···Dy cage. **e** Cage mode in the Dy/O/N core. **f** Coordinated mode of **L**^**1**^.

The nanocluster **Dy**_**30**_ was determined via single-crystal X-ray diffraction. The nanocluster **Dy**_**30**_ crystallized in the trigonal *P*-3*c*1 space group (Fig. 2a and Supplementary Table 2), which contained 30 Dy(III) ions, 12 (H_2_**L**^1^)^4−^ ligands, 4 OH^−^, 36 OAc^−^, 12 H_2_O, and 2 free OH^−^. The size of the nanocluster **Dy**_**30**_ was 2.8 nm × 2.7 nm and the cavity diameter was 1.5 nm (Fig. 2b). The periphery of the Dy/O/N **Dy**_**30**_ core was covered by 12 (H_2_**L**^1^)^4−^ ligands and 36 OAc^−^, forming the structure that enabled the organic ligands to protect the Dy/O/N core of **Dy**_**30**_ (Fig. 2c, d). Each Dy(III) ion in the four triangular lattices Dy_3_ was connected by a folding linear Dy_3_ through ‒N-N‒ bridging on the ligand to form a **Dy**_**30**_ core composed of four triangular Dy_3_ and sixfold linear Dy_3_ units (Fig. 2e). The Dy/O/N core of **Dy**_**30**_ exhibited a spherical cage shape and differed from the octagonal-prismatic **Dy**_**17**_, capsule-shaped **Dy**_**24**_, ball-and-stick-shaped **Dy**_**27**_, and vertex angle cage-shaped **Dy**_**26**_ (or **Dy**_**104**_) (Fig. 2e)^54–57^. In the core of **Dy**_**30**_, the bond distances of Dy···Dy were within the limits of 3.895–3.925 Å in the triangular lattice Dy_3_. The bond distances of Dy···Dy were within the limits of 3.774–3.812 Å in the folding linear Dy_3_ (Supplementary Fig. 3c), and the bond distances of Dy···Dy between triangular lattice Dy_3_ and folding linear Dy_3_ were 5.654–5.717 Å. The ligand in the **Dy**_**30**_ core showed one coordination mode in the structure: *μ*_5_-*η*^1^:*η*^1^:*η*^2^:*η*^2^:*η*^1^:*η*^1^:*η*^2^:*η*^1^:*η*^1^ (Fig. 2f). Structural figure of **Dy**_**30**_ with probability ellipsoids in Supplementary Fig. 5. The thermogravimetric analysis (TGA) curves of **Dy**_**60**_ and **Dy**_**30**_ are discussed in Supplementary Note 2 and shown in Supplementary Fig. 6. The phase purity of **Dy**_**60**_ and **Dy**_**30**_ was examined from their powder X-ray diffraction (PXRD) patterns (Supplementary Note 3 and Supplementary Fig. 7).

SQUEEZE calculations were performed to determine the electron counts per unit cells (Supplementary Note 1).

### Assembly mechanism analysis

Coordination molecule clusters are usually synthesized under hydrothermal/solvothermal conditions at a certain temperature and pressure. Given the temperature and pressure, the tracking and exploration of the self-assembly mechanism were extremely difficult. The outermost electron orbitals of the metal center had clusters of coordination molecules of unpaired electrons and self-assembly was difficult to track via nuclear magnetic resonance spectroscopy (NMR)^48–51^. In recent years, our group used HRESI-MS to track the formation of several lanthanide clusters and proposed their assembly mechanism. For example, the assembly mechanism of Dy-exclusive clusters **Dy**_**3**_, **Dy**_**4**_, **Dy**_**10**_, and **Dy**_**12**_ was proposed^20,21,48–50^. On the basis of the above work, we used HRESI-MS to track the self-assembly of the double-cage-shaped dysprosium cluster **Dy**_**60**_ and its interphase **Dy**_**30**_. The self-assembly of the coordination cage molecular cluster **Dy**_**60**_ and intermediate **Dy**_**30**_ was further inferred by analyzing the species of fragments existing in the solution in different time periods, the time-dependent intensity variation, and the abundance of fragments in the instrument. This work was the first to systematically study the self-assembly mechanism cage coordination molecular clusters. We set the specific time interval in the reaction to remove the same amount of solution from the reaction system, further diluting each equal amount of reaction solution with the same proportion of chromatographic methanol. Data on the anion and cationic modes were collected via HRESI-MS and analyzed.

**Fig. 3:**
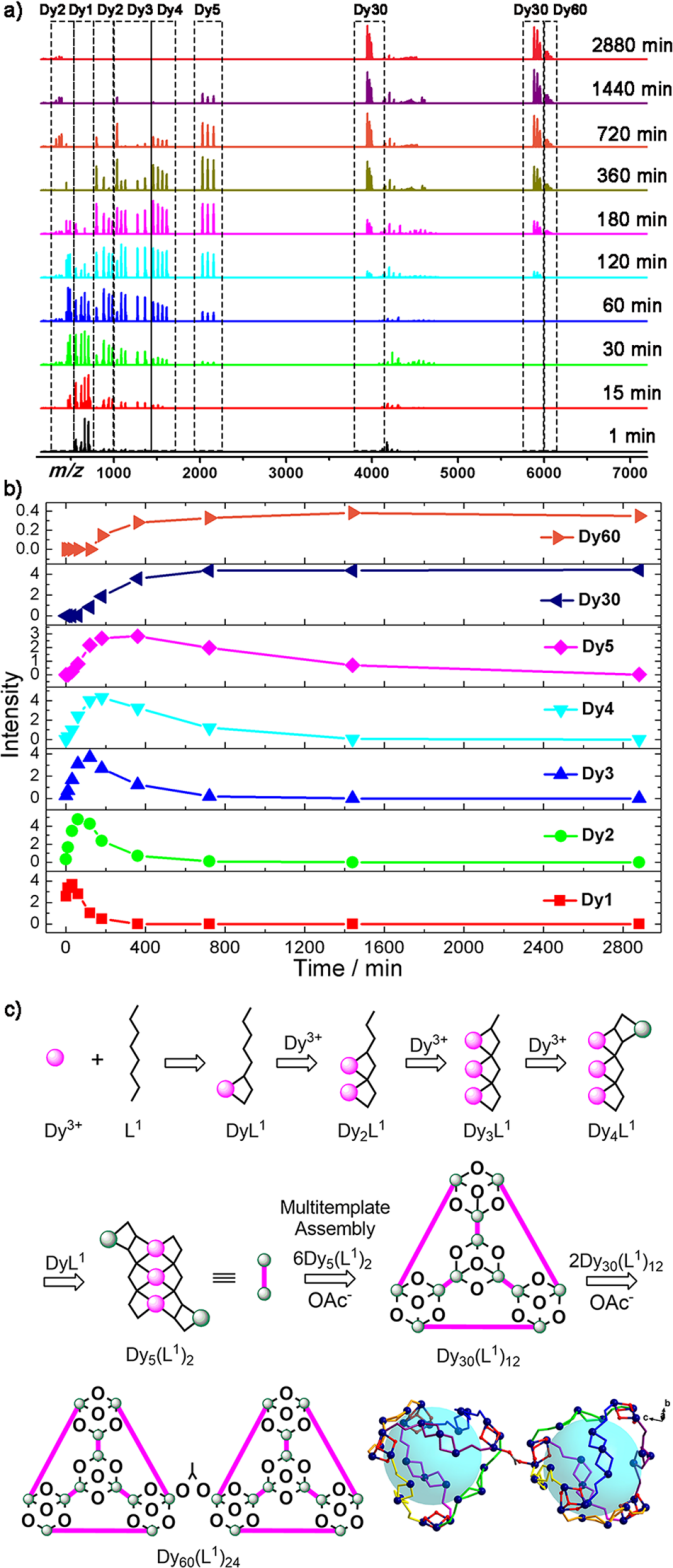
Assembly mechanism analysis of Dy_60_. **a** Time-dependent assembly of cage-shaped **Dy**_**60**_ in cationic mode. **b** HRESI-MS spectra intensity–time profiles of the species in the assembly. **c** Possible assembly mechanism for **Dy**_**60**_.

The data collected in the cationic mode in different time periods of cluster **Dy**_**60**_ formation are shown in Fig. 3a. We propose a plausible assembly mechanism, which is consistent with our data (Fig. 3b). The most reasonable assembly mechanism of **Dy**_**60**_ is shown in Fig. 3c. The HRESI-MS data of **Dy**_**60**_ assembly were analyzed and the results suggested that unit Dy_5_ was obtained via step assembly. A cage-like **Dy**_**30**_ was formed via step-by-step and multi-template assembly, and the two molecules of **Dy**_**30**_ were bridged by OAc^−^ to obtain **Dy**_**60**_. Before heating (0–1 min), the monocyte Dy**L**^1^ (Dy_1_) was the main intensity (*m/z* = 558.07–758.18). The molecular formula of the reaction system was [Dy(H_*x*_**L**^1^)(OAc)_*y*_(solv.)]^+^ (*x* ≤ 2, *y* ≤ 2, *x* + *y* = 2). After heating for 30 min, the content of Dy**L**^1^ in the reaction system decreased gradually for 3 h. The low-strength Dy_2_**L**^1^ (Dy_2_) fragment (*m/z* = 447.54–491.56, 882.07–983.12) appeared in the period of 0–30 min. The molecular formula of the binuclear fragment was [Dy_2_(H_x_**L**^1^)(OAc)_*y*_(solv.)]^+^ (*y* ≤ 2, 2 ≤ *x* ≤ 4, *x* + *y* = 5) or [Dy_2_(H_*x*_**L**^**1**^)(OAc)_*y*_(solv.)]^2+^ (*y* ≤ 2, 2 ≤ *x* ≤ 4, *x* + *y* = 4). With prolonged reaction time, the intensity of Dy_2_**L**^**1**^ was the highest level in 1 h, thereby indicating that the Dy_2_**L**^**1**^ fragment was obtained by chelating Dy^3+^ ion under solvothermal conditions. Dy_2_**L**^**1**^ fragments decreased gradually from 1 h to 12 h. With the further progress of self-assembly, the molecular ion peak of the fragment Dy_3_**L**^**1**^ (Dy_3_) appeared in the range of *m/z* = 1085.97–1362.15 positions. The general formula of the fragments [Dy_3_(H_*x*_**L**^**1**^)(OAc)_*y*_(solv.)]^+^ (*y* ≤ 4, *x* ≤ 2, *x* + *y* = 8) was obtained by fitting. At a reaction time of 2 h, the Dy_3_**L**^**1**^ fragment reached the highest intensity. The intensity of the reaction continued to decrease gradually and disappeared after 24 h. As such, the binuclear fragment Dy_2_**L**^**1**^ continued to capture Dy^3+^ ions to form Dy_3_**L**^**1**^ fragments during this time period. At 15 min of the reaction, the molecular ion peaks produced by Dy_4_**L**^**1**^ (Dy_4_) fragments appeared in the range of *m/z* = 798.53 and 1457.96–1613.08, and the general formulas were obtained by fitting [Dy_4_(H_*x*_**L**^**1**^)(OAc)_*y*_(solv.)]^+^ (*y* ≤ 4, *x* ≤ 2, *x* + *y* = 8). The intensity of the above Dy_4_**L**^**1**^ fragments increased with prolonged reaction time and reached the highest intensity at 3 h, indicating that the Dy_4_**L**^**1**^ fragments were continuously produced in the reaction system. At a reaction time of 30 min, the molecular ion peak of Dy_5_(**L**^**1**^)_2_ (Dy_5_) appeared in the range of *m/z* = 2031.03–2160.10 and the general formulas of [Dy_5_(H_2_**L**^**1**^)_2_(OAc)_*y*_(O)_*z*_(solv.)]^+^ (*z* ≤ 1, 5 ≤ *y* ≤ 6, *y* + *z* = 6) were obtained by fitting. The intensity of the above Dy_5_(**L**^**1**^)_2_ fragments reached its highest level in 6 h and decreased rapidly thereafter. Thus, a large number of Dy_5_(**L**^**1**^)_2_ fragments was used for the next assembly. After 1 h, a small amount of molecular ion peak Dy_30_(**L**^**1**^)_12_ (**Dy**_**30**_) appeared in the range of *m/z* = 3900–6000 and its molecular formula was [Dy_30_(H_2_**L**^**1**^)_12_(OAc)_35_(OH)_4_(solv.)]^3+^ or [Dy_30_(H_2_**L**^**1**^)_12_(OAc)_35_(OH)_3_(solv.)]^4+^. With prolonged heating time, the intensity of Dy_30_(**L**^**1**^)_12_ gradually increased to the highest level. The highest level was reached from 12 h, indicating that the multimolecular Dy_5_(**L**^**1**^)_2_ fragment was assembled by template to form the Dy_30_(**L**^**1**^)_12_ fragment. With continuous reaction for 3 h, the molecular ion peak of Dy_60_(**L**^**1**^)_24_ (**Dy**_**60**_) appeared in the range of *m/z* = 6000–6100 and their molecular formulas were [Dy_60_(H_2_**L**^**1**^)_24_(OAc)_71_(OH)_6_(solv.)]^2+^. With prolonged time, the abundance of the above fragment increased gradually, but the overall strength of the fragments was not high. The structure of the final cluster was compared, and the fragment was a Dy_60_(**L**^**1**^)_24_ fragment frame formed by two Dy_30_(**L**^**1**^)_12_ fragments bridged by an OAc^−^ bridge. However, the abundance of the fragment was not high under the condition of HRESI-MS test, because the OAc^−^ connection was unstable. Analysis of the fragment abundance and changing trend in cationic mode indicated that the cage coordination molecular cluster Dy_60_ formed via step-by-step and multi-template assembly (Supplementary Table 3 and Supplementary Fig. 8). Combined with structural analysis, the possible assembly mechanism is as follows: H_6_**L**^**1**^ → Dy**L**^**1**^ → Dy_2_**L**^**1**^ → Dy_3_**L**^**1**^ → Dy_4_**L**^**1**^ → Dy_5_(**L**^**1**^)_2_ → Dy_30_(**L**^**1**^)_12_ → Dy_60_(**L**^**1**^)_24_ (Fig. 3c). This is a possible assembly mechanism indicated by the time-dependent HRESI-MS tracking cluster **Dy**_**60**_ formation process. The main frame peak appeared at the *m/z* = 399.98 positions in the negative mode and the molecular fragment was [Dy(OAc)_4_]^−^ (calc. 399.98) by fitting (Supplementary Figs. 9 and 10). To further verify the above assembly mechanism, we have tried to volatilize the reaction solution at different time periods, but have not obtained the structure of the intermediate. As for the fragments Dy_1_ ~ Dy_5_ are intermediates of **Dy**_**60**_ formation process, the above intermediates are very unstable in solution and have high Gibbs free energy, so we think it is difficult for them to remain in the reaction solution for a long time and crystallize.

**Fig. 4:**
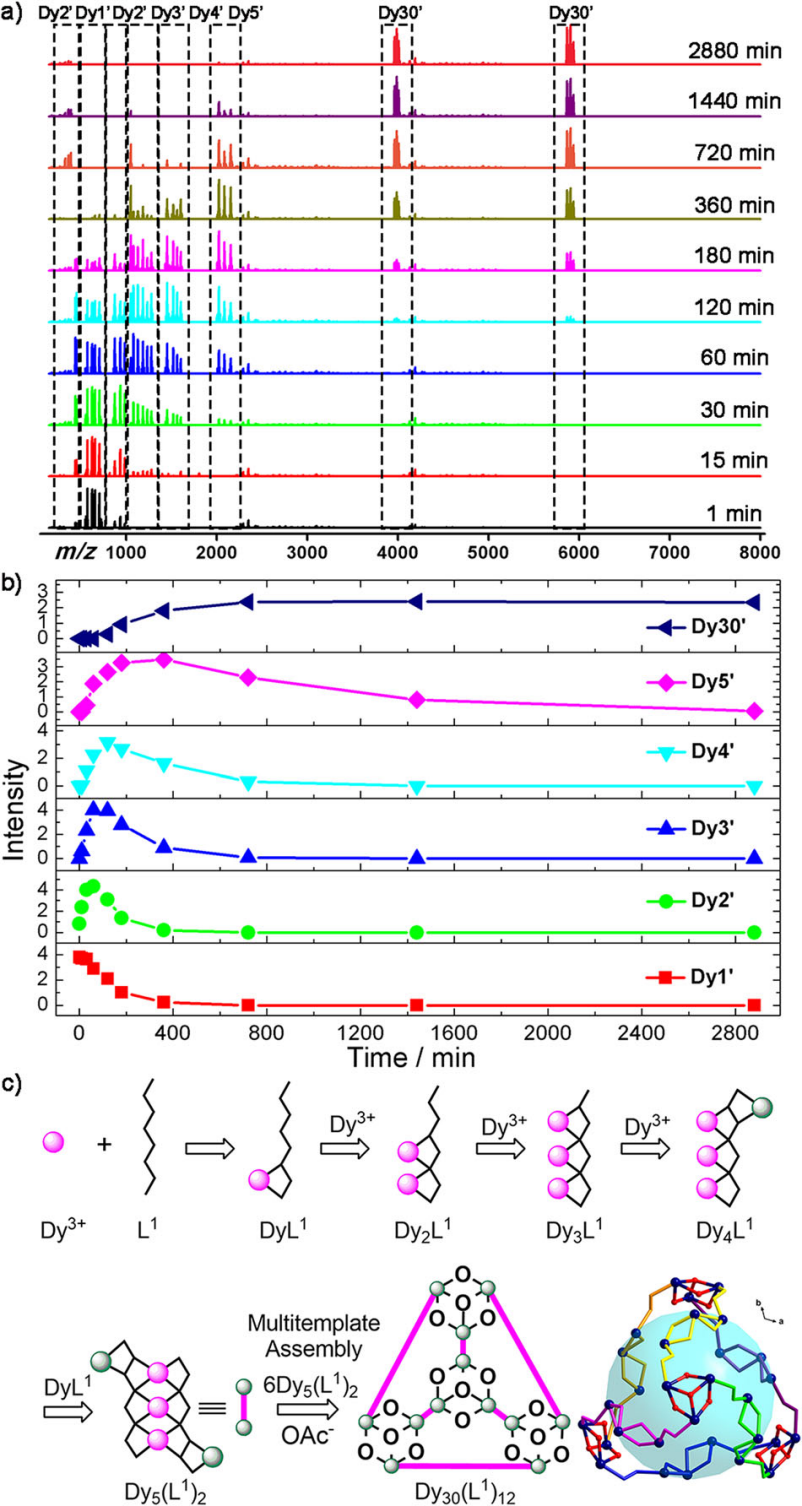
Assembly mechanism analysis of Dy_30_. **a** Time-dependent HRESI-MS of cage-shaped **Dy**_**30**_ in cationic mode. **b** HRESI-MS spectra intensity–time profiles of the species. **c** Possible assembly mechanism of **Dy**_**30**_.

A cage-shaped coordination molecule cluster **Dy**_**30**_ was successfully obtained by replacing Bu_4_NOH in the reaction system with LiOH and reacting under the same conditions to verify the accuracy of **Dy**_**60**_ assembly mechanism. The **Dy**_**30**_ structure described above verified the mechanism of **Dy**_**60**_ assembly. We also used HRESI-MS to track the **Dy**_**30**_ reaction. As shown in Fig. 4a, the variation trend of the time dependence of the molecular ion peak intensity of each component was plotted in Fig. 4b. The most reasonable assembly mechanism of the single cage-shaped coordination molecular cluster **Dy**_**30**_ was estimated in Fig. 4c. The HRESI-MS data for the analysis of the assembly of the cage-shaped coordination molecule cluster **Dy**_**30**_ were similar to that of **Dy**_**60**_ and the self-assembly process was first subjected to step-by-step assembly to form a construction unit. Finally, the final cage-shaped coordination molecular cluster **Dy**_**30**_ was formed via one-step multi-template assembly. The reaction system was the main strength (*m/z* = 576.07–752.18) and the molecular formulas were [Dy(H_*x*_**L**^**1**^)(CH_3_O)_*y*_(OH)_*z*_(solv.)]^+^ (*x* ≤ 2, *y* ≤ 1, *z* ≤ 1, *x* + *y* + *z* = 2). The intensity of Dy**L**^**1**^ in the further reaction system gradually decreased after 15 min of the reaction. The Dy_2_**L**^**1**^ (Dy_2_′) fragment (*m/z* = 440.54–454.58, 887.07–987.12) with low intensity appeared for the time period from 0 min to 15 min. The molecular formula of the Dy_2_**L**^**1**^ fragments obtained by fitting were [Dy_2_(H**L**^**1**^)(OAc)_*y*_(CH_3_O)_*z*_(solv.)]^+^ (*y* ≤ 2, *z* ≤ 1, *y* + *z* = 2) or [Dy_2_(H_*x*_**L**^**1**^)(OAc)_*y*_(solv.)]^2+^ (*y* ≤ 2, 3 ≤ *x* ≤ 4, *x* + *y* = 5). During the reaction, the abundance of the Dy_2_**L**^**1**^ fragment reached the highest at 30 min and gradually decreased from 30 min and almost disappeared after 6 h. The reaction continued and the molecular ion peak of Dy_3_**L**^**1**^ (Dy_3_′) appeared in the range of *m/z* = 1085.97–1362.15, and the molecular formula of the fragment peaks was determined by fitting to [Dy_3_(H_*x*_**L**^**1**^)(OAc)_*y*_(solv.)]^+^ (*y* ≤ 4, *x* ≤ 2, *x* + *y* = 8). At 1 h of reaction time, the fragment Dy_3_**L**^**1**^ reached the highest intensity and the intensity of the reaction decreased gradually and disappeared after 6 h. A certain amount of Dy_4_**L**^**1**^ (Dy_4_′) fragment was present in the solution after 15 min and its molecular ion peaks appeared in the range of *m/z* = 1453.97–1604.08. The molecular formulas of [Dy_4_(H_2_**L**^**1**^)(OAc)_*y*_(O)_*z*_(solv.)]^+^ (*y* ≤ 6, *z* ≤ 1, *y* + *z* = 6) were obtained by fitting. The intensity of the Dy_4_**L**^1^ fragments was increased at the end of the reaction and reached the highest intensity in 2 h. The molecular ion peaks of Dy_5_(**L**^1^)_2_

(Dy_5_’) appeared in the range of *m/z* = 2026.03–2156.10 and the molecular formulas of [Dy_5_(H_2_**L**^1^)_2_(OAc)_*y*_(O)_*z*_(solv.)]^+^ (*z* ≤ 1, 5 ≤ *y* ≤ 6, *y* + *z* = 6) were obtained by fitting. The intensity of the Dy_5_(**L**^**1**^)_2_ fragments peaked in 3 h. The Dy_5_(**L**^**1**^)_2_ fragment was rapidly involved in the next reaction. From 2 h, a molecular ion peak of Dy_30_(**L**^**1**^)_12_ (**Dy**_**30**_) appeared in *m/z* = 3900–6000 and the molecular formulas of [Dy_30_(H_2_**L**^**1**^)_12_(OAc)_35_(OH)_4_(solv.)]^3+^ or [Dy_30_(H_2_**L**^**1**^)_12_(OAc)_35_(OH)_3_(solv.)]^4+^ were obtained by fitting. With prolonged heating time, the intensity of Dy_30_(**L**^**1**^)_12_ gradually increased and peaked, starting from 12 h to the highest. The segments Dy_5_(**L**^**1**^)_2_ formed Dy_30_(**L**^**1**^)_12_ fragments by template assembly during this time period (Supplementary Table 4 and Supplementary Fig. 11). With prolonged treatment, the Dy_60_(**L**^**1**^)_24_ fragment frameworks did not exhibit a high *m/z* range, indicating that LiOH added under these reaction conditions blocked the reaction; thus, stable intermediate segments Dy_30_(**L**^**1**^)_12_ were obtained. In conclusion, the assembly mechanism of the cage-shaped coordination molecular cluster **Dy**_**30**_ was found by analyzing the type of the fragment in the cation mode and its changing tendency as follows: H_6_**L**^**1**^ → Dy**L**^**1**^ → Dy_2_**L**^**1**^ → Dy_3_**L**^**1**^ → Dy_4_**L**^**1**^ → Dy_5_(**L**^**1**^)_2_ → Dy_30_(**L**^**1**^)_12_ (Fig. 4c). This is a possible assembly mechanism proposed by time-dependent HRESI-MS tracking cluster **Dy**_**30**_ formation process. No molecular ion peak fragment with cluster nuclei >5 was found in the reaction system, so we speculate that **Dy**_**30**_ may be formed by Dy_5_(**L**^**1**^)_2_ undergoing template assembly. The main frame peak appeared at the *m/z* = 399.98 position in the negative mode and the molecular fragment was [Dy(OAc)_4_]^−^ (calc. 399.98) by fitting (Supplementary Figs. 12 and 13). The above results demonstrated the self-assembly mechanism of the maximum double-cage-shaped **Dy**_**60**_. We also used HRESI-MS to verify the stability of all complexes in methanol; **Dy**_**30**_ is stable in the solution, which is in good agreement with the time-dependent process. Further, for **Dy**_**60**_ cluster, it does exist mainly as **Dy**_**30**_ fragments in the solution, but there is also some fragment peak of the main frame **Dy**_**60**_ (Supplementary Figs. 14 and 15).

The direct-current magnetic susceptibilities of **Dy**_**30**_ and **Dy**_**60**_ were tested from 300 K to 2 K under a 1000 Oe field (Supplementary Fig. 16). At 300 K, the *χ*_m_*T*-values were 421.88 and 842.s76 cm^3^ K mol^−1^ for **Dy**_**30**_ and **Dy**_**60**_, respectively, which were closed to the isolated values for 30 and 60 Dy(III) ions (^6^*H*_15/2_, *g* = 4/3). Upon cooling, the *χ*_m_*T*-values were marginally decreased up to ca. 100 K before going through shallow minima around 50 K. As the temperature decreased, *χ*_m_*T* decreased to 190.39 and 325.77 cm^3^ K mol^−1^ at 2 K for **Dy**_**30**_ and **Dy**_**60**_. *χ*_m_^−1^ vs. *T* data above 100 K were analyzed using Curie–Weiss law and the obtained Curie constants (*C*) were 431.03 and 854.70 cm^3^ K mol^−^^1^. The Weiss constants (*θ*) were −7.70 K and −5.13 K (Supplementary Fig. 17)^8^. The *χ*_m_*T* was rapidly decreasing in the low-temperature region probably attributed to thermal depopulation in Stark sublevels together with their different magnetic interactions. This decrease is mainly due to the thermal depopulation of the excited *m*_J_ states of Dy(III) and/or the magnetic interactions.

The isothermal magnetization (*M*) of **Dy**_**30**_ and **Dy**_**60**_ were measured at different temperatures (Supplementary Fig. 18)^8^. The magnetization of **Dy**_**30**_ was saturation for at 50 kOe and it reaches a maximum of 104.01 *N*_μB_ at 50 kOe measured at 2 K. However, the absence of saturation for the magnetization of **Dy**_**60**_ even at 50 kOe and it reaches a maximum of 206.82 *N*_μB_ at 50 kOe measured at 2 K. We study their slow magnetic relaxation behaviors and the dynamic magnetic susceptibility of temperature dependence of **Dy**_**30**_ and **Dy**_**60**_. The temperature-dependent *χ*′ and *χ*″ ac susceptibilities of **Dy**_**30**_ and **Dy**_**60**_ were measured from 2 to 12 K under 0 Oe at 1, 10, 499, 700, and 999 Hz, respectively (Supplementary Fig. 19). Magnetic tests showed that both **Dy**_**30**_ and **Dy**_**60**_ exhibited significant single-molecule magnet behavior. For further understanding of the relaxation process of **Dy**_**30**_ and **Dy**_**60**_, Cole–Cole diagrams derived from frequency-dependent ac susceptibilities were drawn (Supplementary Figs. 20 and 21). The Cole–Cole plots followed a single relaxation Debye model, which affords the fitting parameters *τ* (relaxation time) and *α* (which determines the width of the distribution of relaxation times). The ln(*τ*/s) vs. *T*^ −1^ curves for **Dy**_**30**_ and **Dy**_**60**_ (Supplementary Figs. 19d and 20d) were also plotted from these fitting results. The temperature-dependent relaxation time was analyzed by assuming a thermally activated process following the Arrhenius law (*τ* = *τ*_0_exp(*U*_eff_/*k*_B_*T*)). In the low temperature, we applied an Orbach relaxation process (*τ*^−1^ = *τ*_0_^−1^exp( − *U*_eff_*/k*_B_*T*)). After performing Orbach fitting on **Dy**_**30**_ and **Dy**_**60**_, we got their *U*_eff_ = 11.15 K and 15.93 K, *τ*_0_ = 7.27 × 10^−6^ s and 1.66 × 10^−6^ s, respectively. In addition, we considered the Orbach and Raman processes (*τ*^−1^ = *τ*_0_^−1^exp(−*U*_eff_*/k*_B_*T*) + *CT*^n^) to fit **Dy**_**30**_ and **Dy**_**60**_ over the full temperature range, we got their *U*_eff_ = 21.74 K and 20.14 K, *τ*_0_ = 2.88 × 10^−6^ s and 1.24 × 10^−6^ s, respectively (Supplementary Figs. 10–21).

In summary, we synthesized the Dy-exclusive double-cage-shaped cluster **Dy**_**60**_ under solvothermal conditions by using a diacylhydrazone ligand with multidentate coordination. **Dy**_**60**_ was formed by two identical **Dy**_**30**_s through a *μ*_2_-OAc^−^ bridge and its cluster core was composed of only two different types of Dy_3_, which were a triangular shape Dy_3_ and a polygonal line type Dy_3_. We changed the base added in the reaction to obtain a single cage-shaped cluster **Dy**_**30**_ with a spherical cavity. To the best of our knowledge, **Dy**_**30**_ is currently the largest Dy-exclusive single cage-shaped cluster. HRESI-MS was used to track the formation of **Dy**_**60**_. Various intermediate fragments were screened and further combined with the changing trend of these intermediate fragments. We proposed the following possible self-assembly mechanism of **Dy**_**60**_: H_6_**L**^**1**^ → Dy**L**^**1**^ → Dy_2_**L**^**1**^ → Dy_3_**L**^**1**^ → Dy_4_**L**^**1**^ → Dy_5_(**L**^**1**^)_2_ → Dy_30_(**L**^**1**^)_12_ → Dy_60_(**L**^**1**^)_24_. Similarly, we tracked the formation of **Dy**_**30**_ and proposed its possible assembly mechanism as follows: H_6_**L**^**1**^ → Dy**L**^**1**^ → Dy_2_**L**^**1**^ → Dy_3_**L**^**1**^ → Dy_4_**L**^**1**^ → Dy_5_(**L**^**1**^)_2_ → Dy_30_(**L**^**1**^)_12_. HRESI-MS did not find molecular ion peak fragments with cluster nuclei >5 in the reaction solution that formed **Dy**_**30**_, so we speculated that **Dy**_**30**_ may be formed by Dy_5_(**L**^**1**^)_2_ undergoing template assembly. The synthesis of the cluster **Dy**_**30**_ and its assembly mechanism further verified the formation process of **Dy**_**60**_. We used crystallography and HRESI-MS combination technology to study the assembly mechanism of the Dy-exclusive double-cage-shaped cluster **Dy**_**60**_ and its intermediate single cage-shaped cluster **Dy**_**30**_. To the best of our knowledge, this work is the first to use HRESI-MS for tracking the formation of cage-shaped Ln(III) clusters. This work provided a set of methods to study the formation tracking and assembly mechanism of high-nuclearity lanthanide clusters. It also lays the foundation for the further design and manipulation of high-nuclearity lanthanide clusters.

## Methods

### Materials and measurements

All reagents were obtained from commercial sources and used without further purification. Elemental analyses for C, H, and N were performed on a vario MICRO cube. TGA analyses were conducted in a flow of nitrogen at a heating rate of 5 °C min^−1^ using a NETZSCH TG 209 F3 (Supplementary Fig. 6). PXRD spectra were recorded on a D8 Advance (Bruker) diffractometer at 293 K (Cu-Kα). The samples were prepared by crushing crystals and the powder placed on a grooved aluminum plate. Diffraction patterns were recorded from 3º to 65º at a rate of 5° min^−1^. Calculated diffraction patterns of the compounds were generated with the Mercury software (Supplementary Fig. 7). Infrared spectra were recorded by transmission through KBr pellets containing ca. 0.5% of the complexes using a PE Spectrum FT-IR spectrometer (400–4000 cm^−1^).

### Single-crystal X-ray crystallography

Diffraction data for these complexes were collected on a Bruker SMART CCD diffractometer (Cu-Kα radiation and *λ* = 1.54184 Å) in *Φ* and *ω* scan modes. The structures were solved by direct methods, followed by difference Fourier syntheses, and then refined by full-matrix least-squares techniques on *F*^2^ using SHELXL^58^. All other non-hydrogen atoms were refined with anisotropic thermal parameters. Hydrogen atoms were placed at calculated positions and isotropically refined using a riding model. Supplementary Table S1 summarizes X-ray crystallographic data and refinement details for the complexes. Full details can be found in the CIF files provided in the Supplementary Information. The Cambridge Crystallographic Data Centre reference numbers are 1947876 (Dy_30_) and 1948703 (Dy_60_).

### HRESI-MS measurement

HRESI-MS measurements were conducted at a capillary temperature of 275 °C. Aliquots of the solution were injected into the device at 0.3 mL h^−1^. The mass spectrometer used for the measurements was a ThermoExactive and the data were collected in positive and negative ion modes. The spectrometer was previously calibrated with the standard tune mix to give a precision of ca. 2 p.p.m. within the region of 200−7500 *m/z*. The capillary voltage was 50 V, the tube lens voltage was 150 V, and the skimmer voltage was 25 V.

### Crystal structure determination

Because of the disorder of the free CH_3_OH and CH_3_CN molecules, parts of the CH_3_OH and CH_3_CN molecules are difficult to locate in the final structural refinement. The number of free molecules is further confirmed by elemental analyses and TGA analysis (Supplementary Fig. 4). Therefore, the chemical formula of complexes are found to be [Dy_30_(H_2_L^1^)_12_(OAc)_36_(OH)_4_(H_2_O)_12_]·2OH·10H_2_O·12CH_3_OH·13CH_3_CN (**Dy**_**30**_) and [Dy_60_(H_2_L^1^)_24_(OAc)_71_(O)_5_(OH)_3_(H_2_O)_27_]·6H_2_O·6CH_3_OH·7CH_3_CN (**Dy**_**60**_). Full details can be found in the CIF files.

### Synthesis of H_6_L^1^

The synthetic route for H_6_L^1^ is presented in Supplementary Fig. 1. Subsequently, the dimethyl 4-hydroxy-1H-pyrazole-3,5-dicarboxylate (2.00 g, 10 mmol) and N_2_H_4_·H_2_O (12.5 mL, 80%) was refluxed in MeOH (180 mL) at 80 ^o^C for 12 h. The 2-hydroxybenzaldehyde (3.05 g, 25 mmol) were then added slowly and the reaction was kept at 80 °C for another 12 h. Upon cooling and filtering, light yellow solid of 4-hydroxy-*N,N'*-bis(2-hydroxybenzylidene)-1H-pyrazole-3,5-dicarbohydrazide (H_6_L^1^) was obtained with a yield of 90% (based on 4-hydroxy-1H-pyrazole-3,5-dicarboxylate). High resolution mass spectrometry (HRMS) (*m*/*z*): [M-H]^−^ calcd. for C_19_H_16_N_6_O_5_, 408.1182; found, 407.1103. Elemental analysis (calcd., found for C_19_H_16_N_6_O_5_): C (55.88, 55.73), H (3.95, 3.88), N (20.58, 20.47). IR spectrum (cm^−1^): 3308 (m), 3142 (s), 3055 (s), 1662 (vs), 1619 (vs), 1586 (vs), 1546 (vs), 1489 (s), 1451 (s), 1370 (m), 1347 (m), 1321 (m), 1271 (vs), 1240 (s), 1196 (s), 1101 (w), 1035 (w), 956 (w), 946 (w), 871 (m), 796 (w), 653 (w), 615 (w), 566 (w), 532 (w), 479 (w), 449 (w). ^1^H NMR (400 MHz, CDCl_3_): δ 14.10 (s, 1H), 12.28 (s, 1H), 11.45 (s, 1H), 11.10 (s, 1H), 10.96 (s, 1H), 8.69 (d, *J* = 17.5 Hz, 2H), 7.55 (s, 1H), 7.46 (s, 1H), 7.32-7.30 (m, 2H), 6.95-6.91 (m, 5H); ^13^C NMR (125 MHz, CDCl_3_): δ 159.2, 158.0, 157.9, 155.1, 149.5, 149.2, 143.6, 132.0, 131.8, 130.4, 129.9, 122.9, 119.8, 119.1, 116.9.

### The synthesis method of Dy_60_ and Dy_30_. Dy_30_

A mixture of Dy(OAc)_3_·4H_2_O (0.5 mmol, 205.8 mg), HL^1^ ligand (0.1 mmol, 40.8 mg), LiOH (0.52 mmol, 12.4 mg), 1.6 mL mixted solvent (CH_3_OH : CH_3_CN = 1:1) were stirred and sealed in a 20 cm-long Pyrex tube and heated at 80 °C for 3 days, then it was taken out and slowly cooled to room temperature. In addition, square yellow crystals were observed (Supplementary Table 5). The yield was about 52% (based on ligand). Elemental analysis (calcd., found for C_338_H_389_Dy_30_N_85_O_172_): C (30.59, 30.43), H (2.95, 2.82), N (8.97, 8.78). IR (KBr, cm^−1^): 3007 (m), 1616 (s), 1546 (s), 1441 (w), 1305 (w), 1197 (m), 1034 (s), 891 (s), 761 (s), 648 (s), 534 (s).

**Dy**_**60**_: A mixture of Dy(OAc)_3_·4H_2_O (0.5 mmol, 205.8 mg), HL^1^ ligand (0.1 mmol, 40.8 mg), tetrabutyl ammonium hydroxide (120 µL), 1.6 mL mixted solvent (CH_3_OH:CH_3_CN = 1 : 1) were stirred and sealed in a 20 cm-long Pyrex tube and heated at 80 °C for 2 days, then it was taken out and slowly cooled to room temperature. In addition, square yellow crystals were observed (Supplementary Table 6). The yield was about 68% (based on ligand). Elemental analysis (calcd., found for C_618_H_615_Dy_60_N_151_O_309_): C (29.87, 29.71), H (2.49, 2.37), N (8.51, 8.39). IR (KBr, cm^−1^): 3008 (m), 1614 (s), 1544 (s), 1443 (w), 1307 (w), 1192 (m), 1036 (s), 893 (s), 761 (s), 646 (s), 537 (s).

## Supplementary information

Supplementary Information

Description of Additional Supplementary Files

Supplementary Data 1

Supplementary Data 2

Supplementary Data 3

## Data Availability

All data used in this manuscript are available from the authors upon reasonable request. The X-ray crystallographic coordinates for structures reported in this article have been deposited at the Cambridge Crystallographic Data Centre (CCDC), under deposition number CCDC 1947876 (**Dy**_**30**_) and 1948703 (**Dy**_**60**_). These data can be obtained free of charge from The Cambridge Crystallographic Data Centre via hyperlink http://www.ccdc.cam.ac.uk/data_request/cifwww.ccdc.cam.ac.uk/data_request/cif.
