# Supplementary material for: Assembly of Dy60 and Dy30 cage-shaped nanoclusters
Source: Commun Chem. 2020 Mar 6;3:30. doi: 10.1038/s42004-020-0276-3 (PMC9814749; doi:10.1038/s42004-020-0276-3)
Supplement: Supplementary file 1 — Supplementary Information [file 42004_2020_276_MOESM1_ESM.pdf]

Supplementary Note 1

SQUEEZE results for these two compounds are as follows:[1]

(1) **Dy_{30}**

```
loop_
   _platon_squeeze_void_nr
   _platon_squeeze_void_average_x
   _platon_squeeze_void_average_y
   _platon_squeeze_void_average_z
   _platon_squeeze_void_volume
   _platon_squeeze_void_count_electrons
   _platon_squeeze_void_content
    1   0.000   0.000 -0.001      25717        2394"
    2   0.612   0.144   0.179        15             3"
    3   0.532   0.144   0.679        15             3"
    4   0.478   0.198   0.093        13             3"
    5   0.720   0.198   0.593        13             3"
    6   0.801   0.280   0.093        13             3"
    7   0.478   0.280   0.593        13             3"
    8   0.667   0.333   0.135        51             6"
    9   0.667   0.333   0.635        51             6"
   10   0.532   0.388   0.179        15             3"
   11   0.856   0.388   0.679        15             3"
   12   0.856   0.468   0.179        15             3"
   13   0.612   0.468   0.679        15             3"
   14   0.198   0.478   0.407        13             3"
   15   0.280   0.478   0.907        13             3"
   16   0.720   0.522   0.093        13             3"
   17   0.801   0.522   0.593        13             3"
   18   0.388   0.532   0.321        15             3"
   19   0.144   0.532   0.821        15             3"
   20   0.144   0.612   0.321        15             3"
   21   0.468   0.612   0.821        15             3"
   22   0.333   0.667   0.365        51             6"
   23   0.333   0.667   0.865        51             6"
   24   0.522   0.720   0.407        13             3"
   25   0.198   0.720   0.907        13             3"
   26   0.280   0.801   0.407        13             3"
```

27	0.522	0.801	0.907	13	3"
28	0.468	0.856	0.321	15	3"
29	0.388	0.856	0.821	15	3"

That is, SQUEEZE gives 2490 electrons/unit cell for the voids, and each formula unit has 2490/4 = 622 electrons (since Z = 4). It is well known that 1 H_2O molecule contains 10 electrons, 1 CH_3CN molecule contains 22 electrons, and a CH_3OH molecule contains 18 electrons. Further combined with elemental analysis and thermogravimetric analysis results (Figure S2a), the molecular formula of **Dy$_{30}$** is calculated to be $[Dy_{30}(H_2\mathbf{L}^{\mathbf{1}})_{12}(OAc)_{36}(OH)_4(H_2O)_{12}]\cdot 2OH\cdot 10H_2O\cdot 12CH_3OH\cdot 13CH_3CN$.

(2) **Dy$_{60}$**

loop_

_platon_squeeze_void_nr

_platon_squeeze_void_average_x

_platon_squeeze_void_average_y

_platon_squeeze_void_average_z

_platon_squeeze_void_volume

_platon_squeeze_void_count_electrons

_platon_squeeze_void_content

1	0.509	-0.241	0.238	5168	322 "
2	0.544	0.241	0.762	5163	321"

That is, SQUEEZE gives 643 electrons/unit cell for the voids, and each formula unit has 643/2 = 322 electrons (since Z = 2). It is well known that 1 H_2O molecule contains 10 electrons, 1 CH_3CN molecule contains 22 electrons, and 1 CH_3OH molecule contains 18 electrons. Further combined with elemental analysis and thermogravimetric analysis results (Figure S2b), the molecular formula of **Dy$_{60}$** is calculated to be $[Dy_{60}(H_2\mathbf{L}^{\mathbf{1}})_{24}(OAc)_{71}(O)_5(OH)_3(H_2O)_{27}]\cdot 6H_2O\cdot 6CH_3OH\cdot 7CH_3CN$.

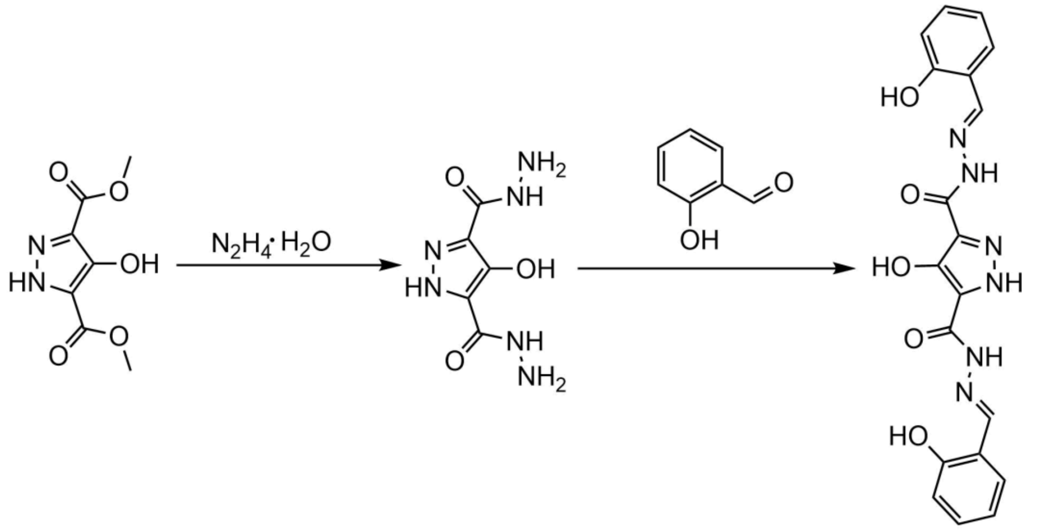

Supplementary Figure 1 Synthetic route of $H_6\mathbf{L}^{\mathbf{1}}$.

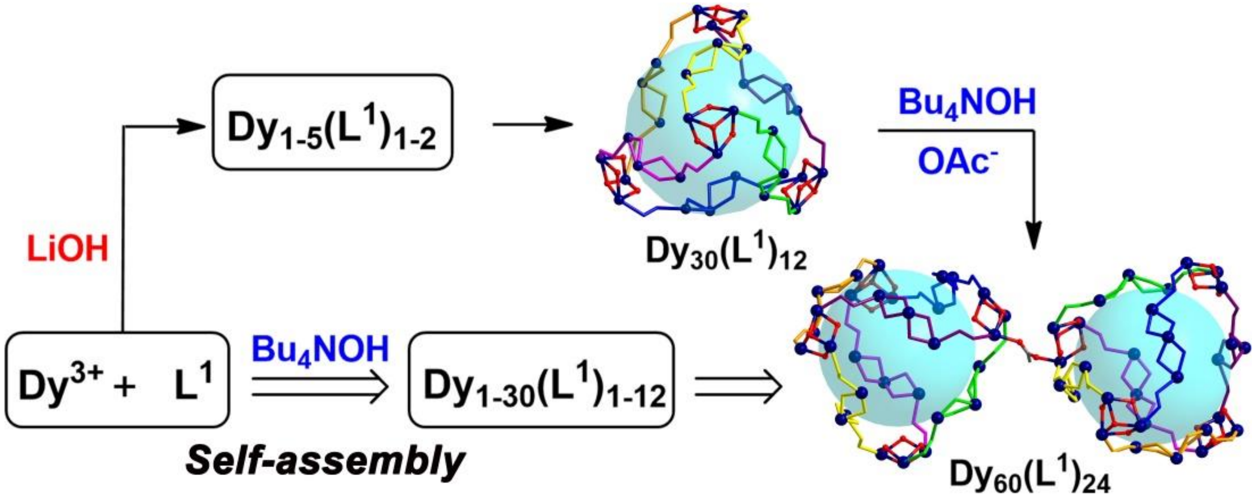

Supplementary Figure 2 Synthesis of the cage-shaped clusters **Dy_{30}** and **Dy_{60}**. Under Bu_4NOH conditions, **Dy_{60}** is finally obtained; under LiOH conditions, **Dy_{30}** is finally obtained.

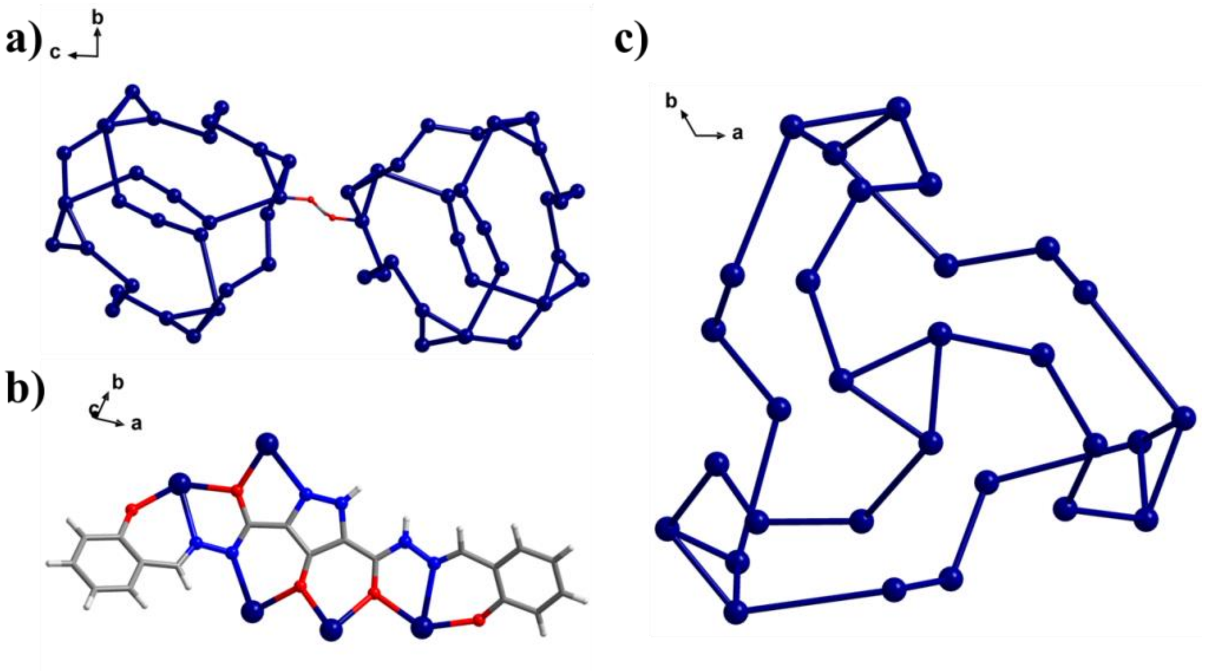

Supplementary Figure 3 a) The bond distances of Dy⋯Dy in **Dy_{60}** core; b) the coordination mode for **Dy_{60}**; c) the bond distances of Dy⋯Dy in **Dy_{30}** core.

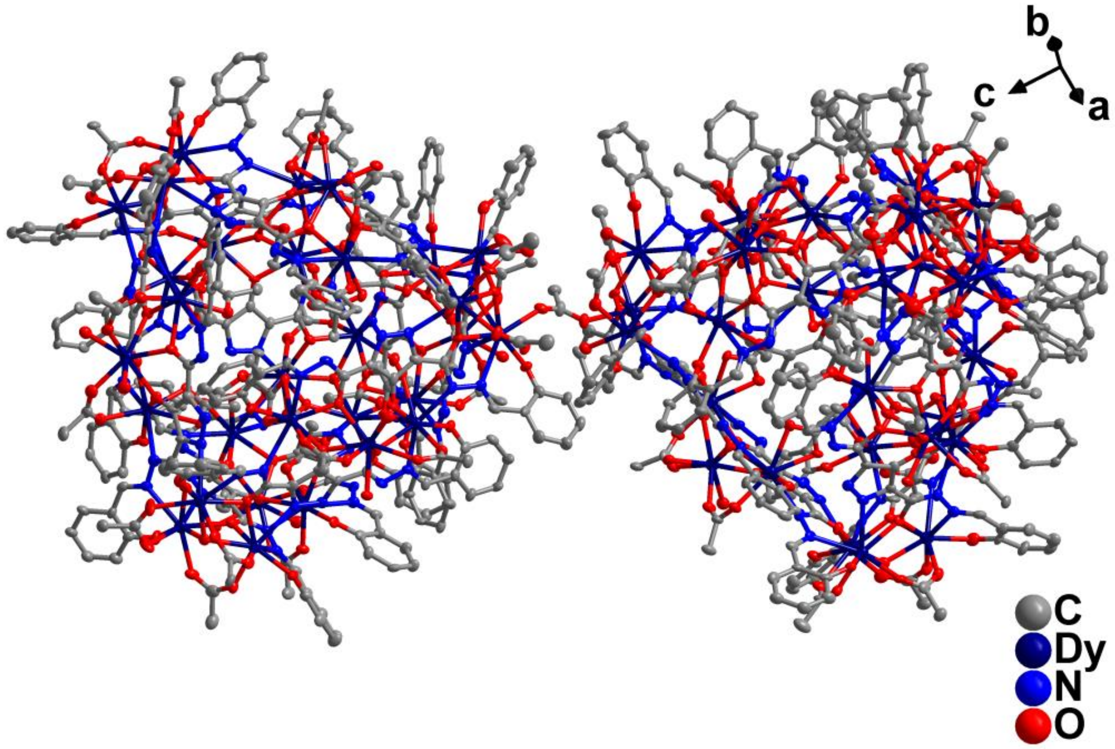

Supplementary Figure 4 Structural figure of **Dy$_{60}$** with probability ellipsoids.

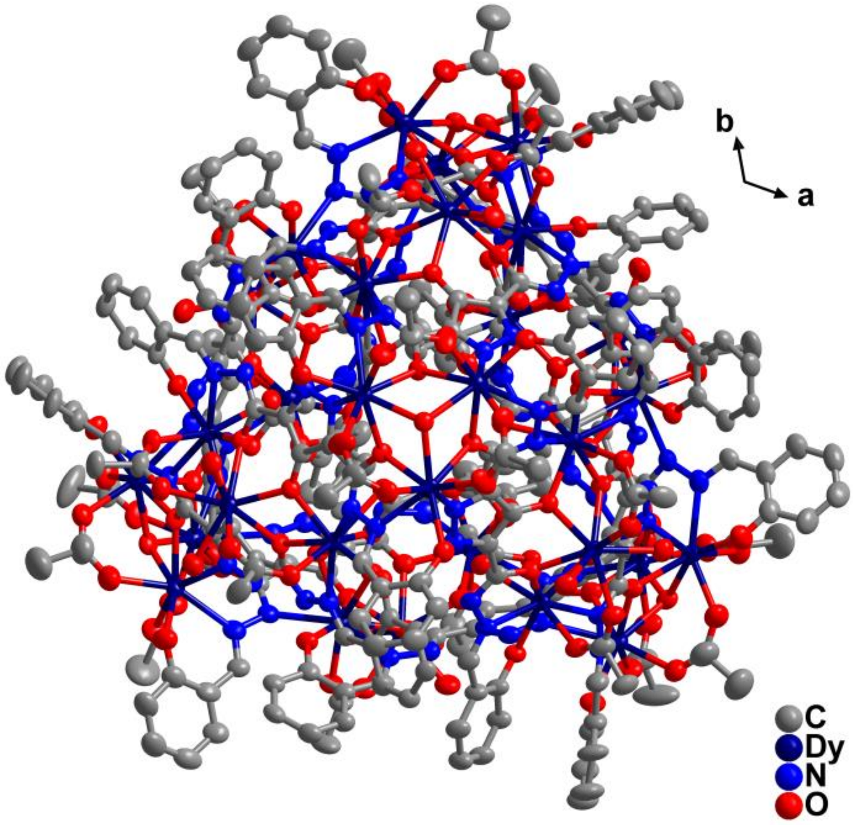

Supplementary Figure 5 Structural figure of **Dy$_{30}$** with probability ellipsoids.

Supplementary Note 2

Thermal analysis. TG data were collected on a Labsys evo TG thermal analyzer under a purge gas of dry nitrogen flowing at 20 mL·min^{-1} and with a heating rate of 5 °C·min^{-1} in the temperature region of 35–1000 °C. **Dy_{30}** and **Dy_{60}** showed remarkable weight loss as the temperature increased from ambient temperature (Supplementary Figure 6). The weight loss of **Dy_{30}** at 35-80 ^{o}C underwent a slow weight loss of 12.03% (calcd 8.53%), which corresponds to the release of thirteen free acetonitrile molecules, twelve free methanol molecules and twelve free water molecules (two waters were from two free hydroxide ion). The second weight loss of 13.78% (calcd 13.54%) in the temperature range of 340–577 °C which could be attributed to the elimination of thirty-six CH_3CO (coming from coordinated acetate radical) and fourteen waters (rooting in twelve coordinated waters and four coordinated hydroxide ion). And the skeleton of **Dy_{30}** began to collapse at 340 °C. The weight loss of **Dy_{60}** (4.93%; calcd 2.36%) occurred at 35–63 °C, which corresponds to the loss of six free water molecules, seven free acetonitrile molecules and six free methanol molecules. The skeleton of **Dy_{60}** began to collapse at temperatures beyond 164 °C.

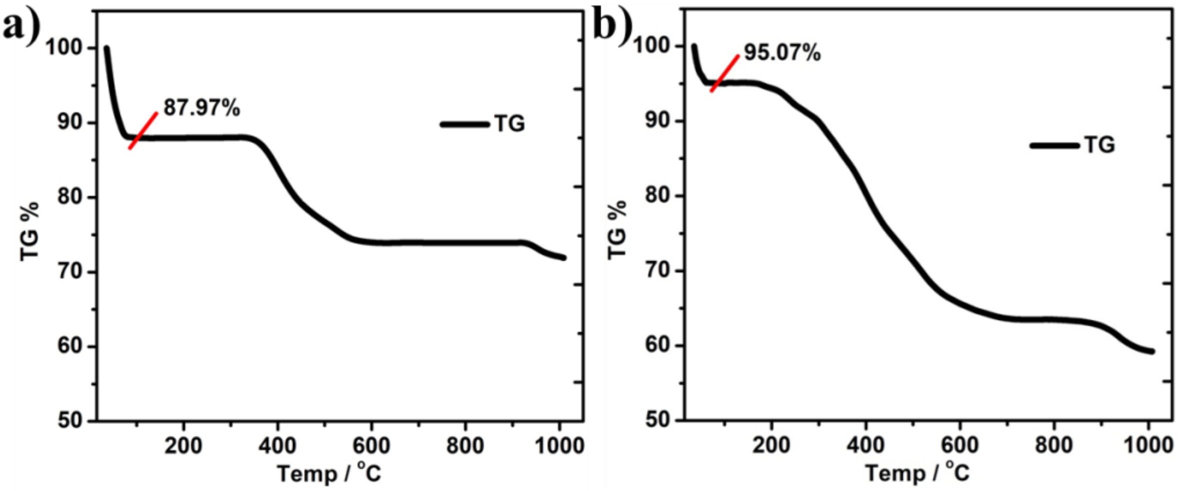

Supplementary Figure 6 The TG curves of **Dy_{30}** (a) and **Dy_{60}** (b) under heating in flowing N_2 at 5 °C·min^{-1} over the temperature range of 35-1000 °C.

Supplementary Note 3

To confirm whether the crystal structures are truly representative of the bulk materials, PXRD experiments were carried out for complex. The PXRD experimental and computer-simulated patterns of the corresponding complex are shown in Supplementary Figure 7. They show that the synthesized bulk materials and the measured single crystals are the similar due to efflorescence of crystals in air.

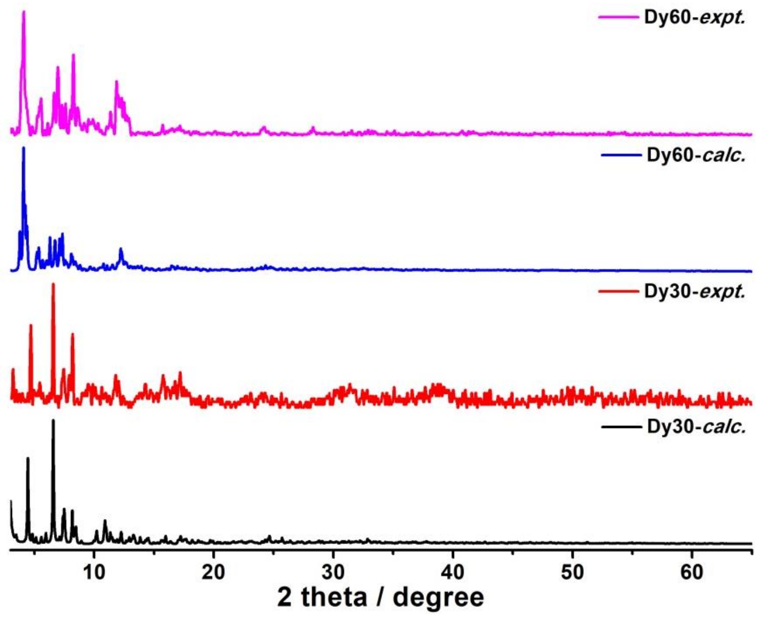

Supplementary Figure 7 Powdered X-ray diffraction (PXRD) patterns for $\mathbf{Dy_{30}}$ and $\mathbf{Dy_{60}}$.

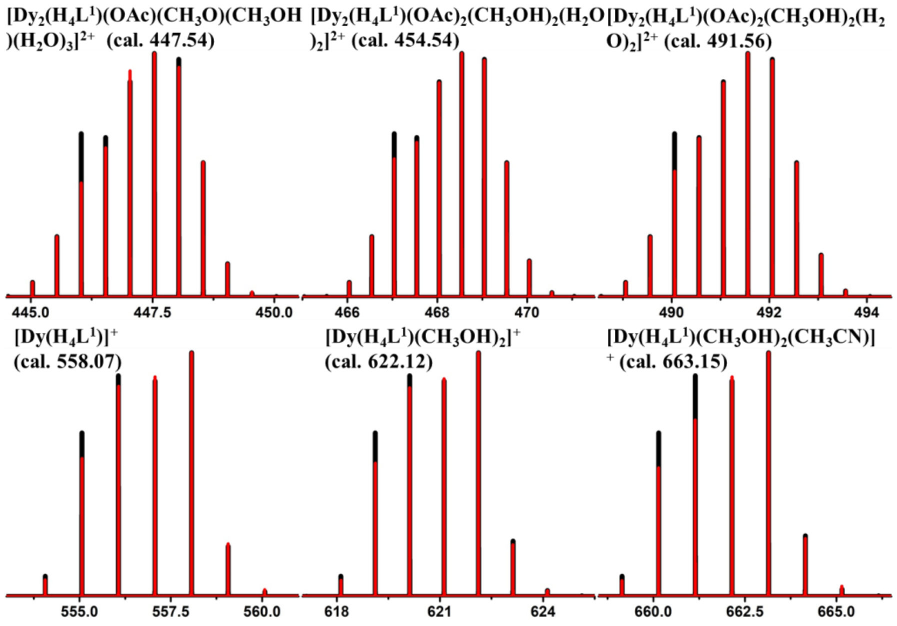

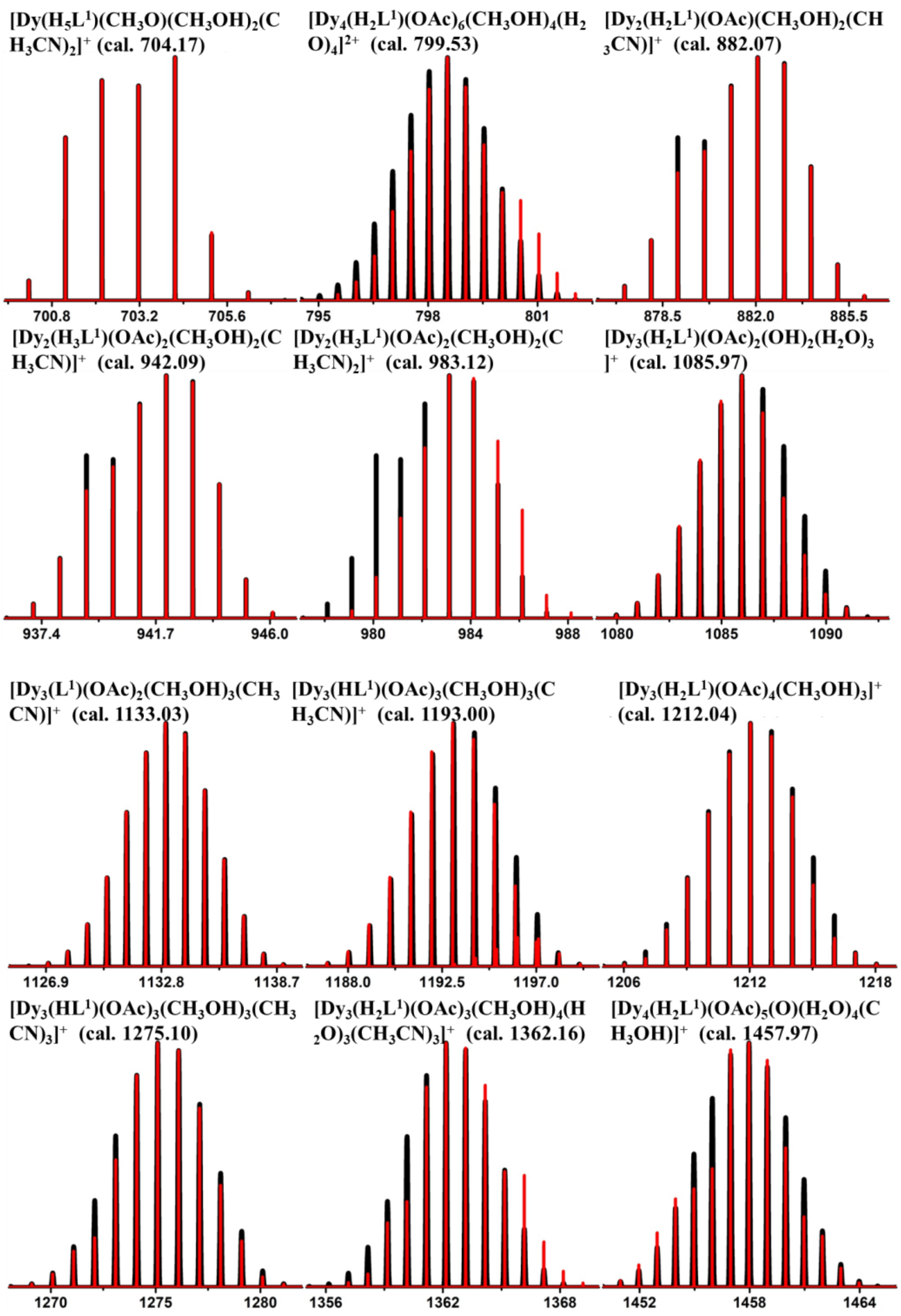

$[Dy(H_5L^1)(CH_3O)(CH_3OH)_2(CH_3CN)_2]^+$ (cal. 704.17)
700.8 703.2 705.6
$[Dy_4(H_2L^1)(OAc)_6(CH_3OH)_4(H_2O)_4]^{2+}$ (cal. 799.53)
795 798 801
$[Dy_2(H_2L^1)(OAc)(CH_3OH)_2(CH_3CN)]^+$ (cal. 882.07)
878.5 882.0 885.5
$[Dy_2(H_3L^1)(OAc)_2(CH_3OH)_2(CH_3CN)]^+$ (cal. 942.09)
937.4 941.7 946.0
$[Dy_2(H_3L^1)(OAc)_2(CH_3OH)_2(CH_3CN)_2]^+$ (cal. 983.12)
980 984 988
$[Dy_3(H_2L^1)(OAc)_2(OH)_2(H_2O)_3]^+$ (cal. 1085.97)
1080 1085 1090
$[Dy_3(L^1)(OAc)_2(CH_3OH)_3(CH_3CN)]^+$ (cal. 1133.03)
1126.9 1132.8 1138.7
$[Dy_3(HL^1)(OAc)_3(CH_3OH)_3(CH_3CN)]^+$ (cal. 1193.00)
1188.0 1192.5 1197.0
$[Dy_3(H_2L^1)(OAc)_4(CH_3OH)_3]^+$ (cal. 1212.04)
1206 1212 1218
$[Dy_3(HL^1)(OAc)_3(CH_3OH)_3(CH_3CN)_3]^+$ (cal. 1275.10)
1270 1275 1280
$[Dy_3(H_2L^1)(OAc)_3(CH_3OH)_4(H_2O)_3(CH_3CN)_3]^+$ (cal. 1362.16)
1356 1362 1368
$[Dy_4(H_2L^1)(OAc)_5(O)(H_2O)_4(CH_3OH)]^+$ (cal. 1457.97)
1452 1458 1464

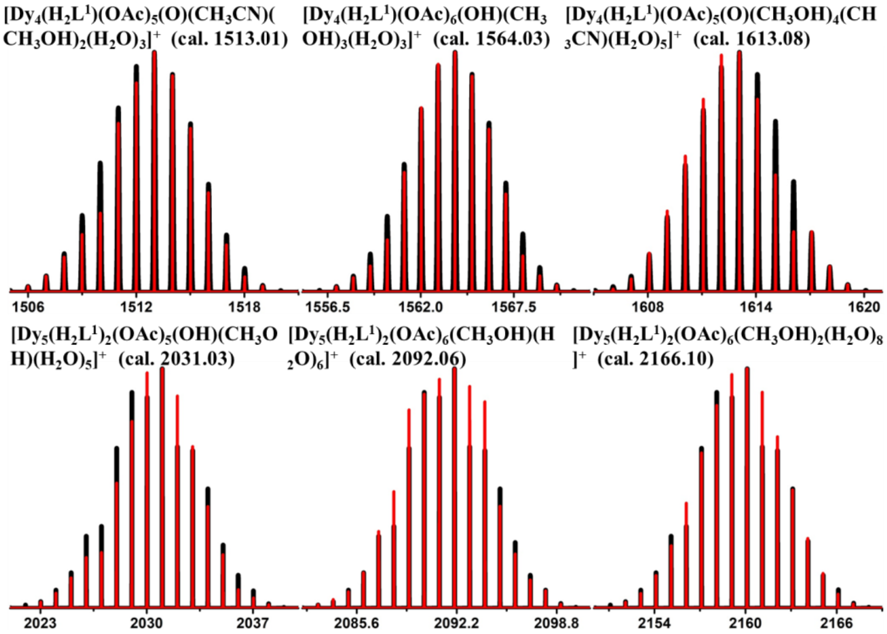

□ = $Dy_{30}(H_2\mathbf{L}^1)_{12}(OAc)_{35}(OH)_4$

1: [□ $(CH_3OH)_3(H_2O)]^{3+}$ (cal. 3944.30)
2: [□ $(CH_3OH)_3(H_2O)_5]^{3+}$ (cal. 3968.63)
3: [□ $(CH_3OH)_4(H_2O)_7]^{3+}$ (cal. 3990.00)
4: [□ $(OH)(H_2O)_2]^{2+}$ (cal. 5884.42)
5: [□ $(OH)(H_2O)_6]^{2+}$ (cal. 5925.45)
6: [□ $(OH)(CH_3OH)_2(H_2O)_6]^{2+}$ (cal. 5953.45)

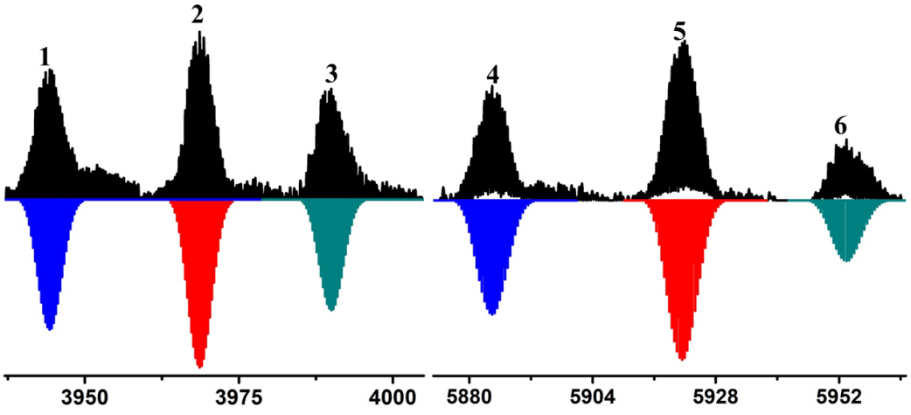

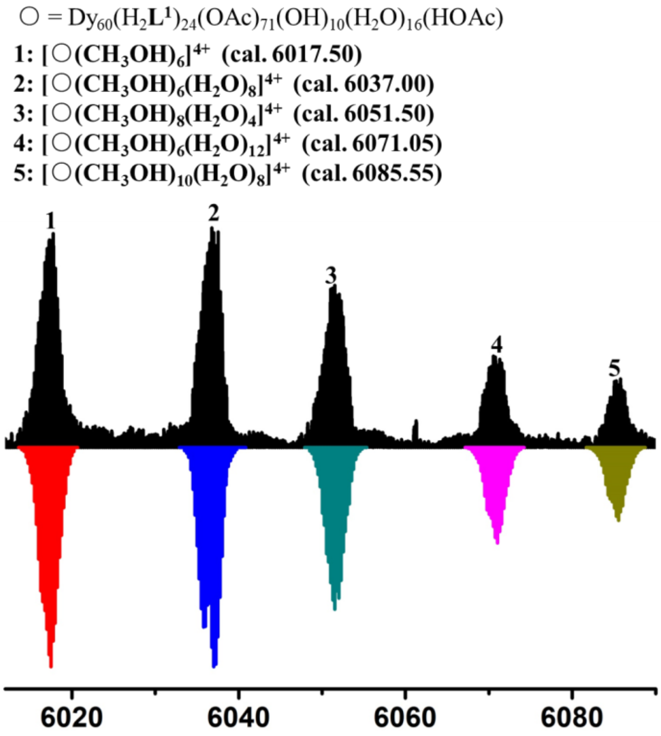

Supplementary Figure 8 The superposed simulated and observed spectra of several species in the time-dependent HRESI-MS of **Dy_{60}** (cation mode).

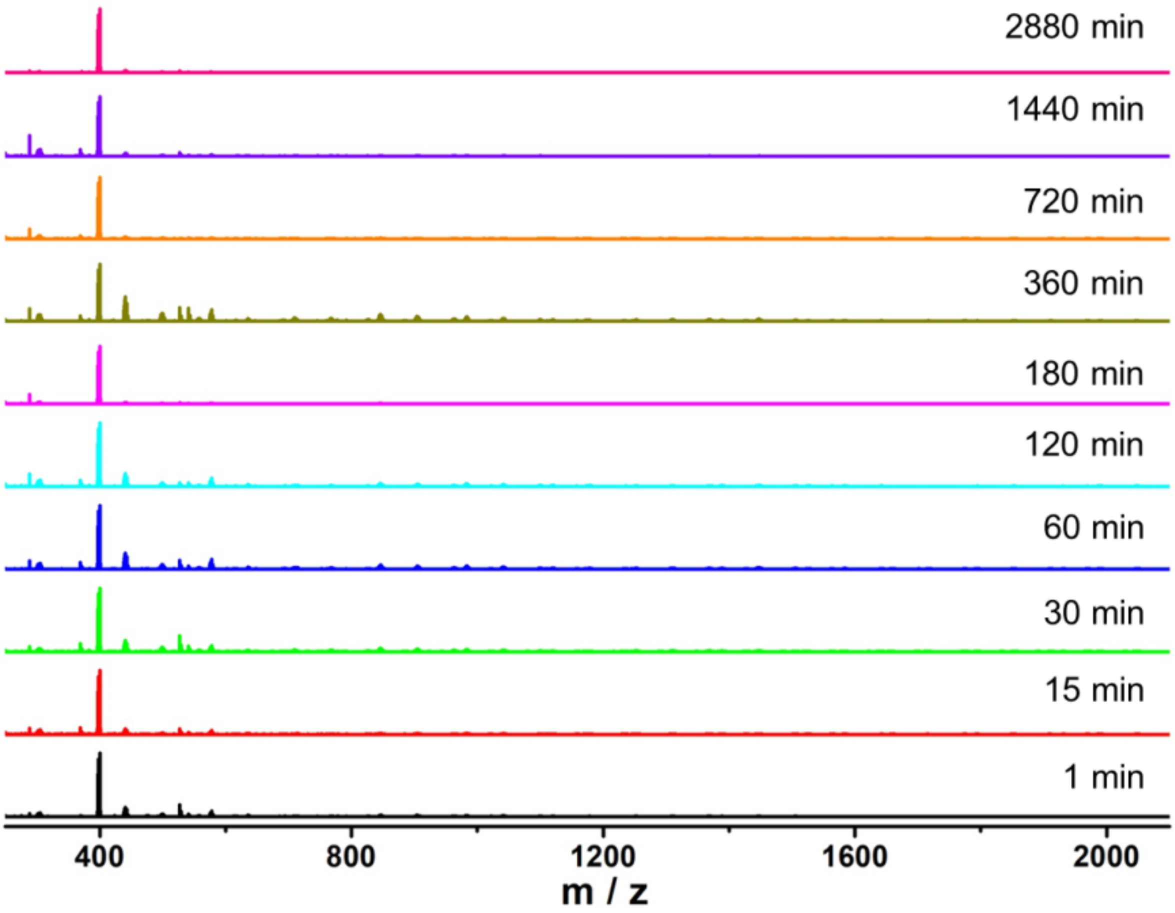

Supplementary Figure 9 Time-dependent HRESI-MS spectra of **Dy_{60}** in negative mode.

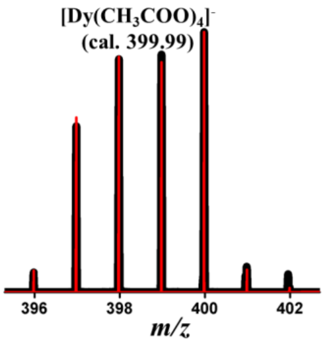

Supplementary Figure 10 The superposed simulated and observed spectra of several species in the time-dependent HRESI-MS of **Dy_{60}** (negative mode).

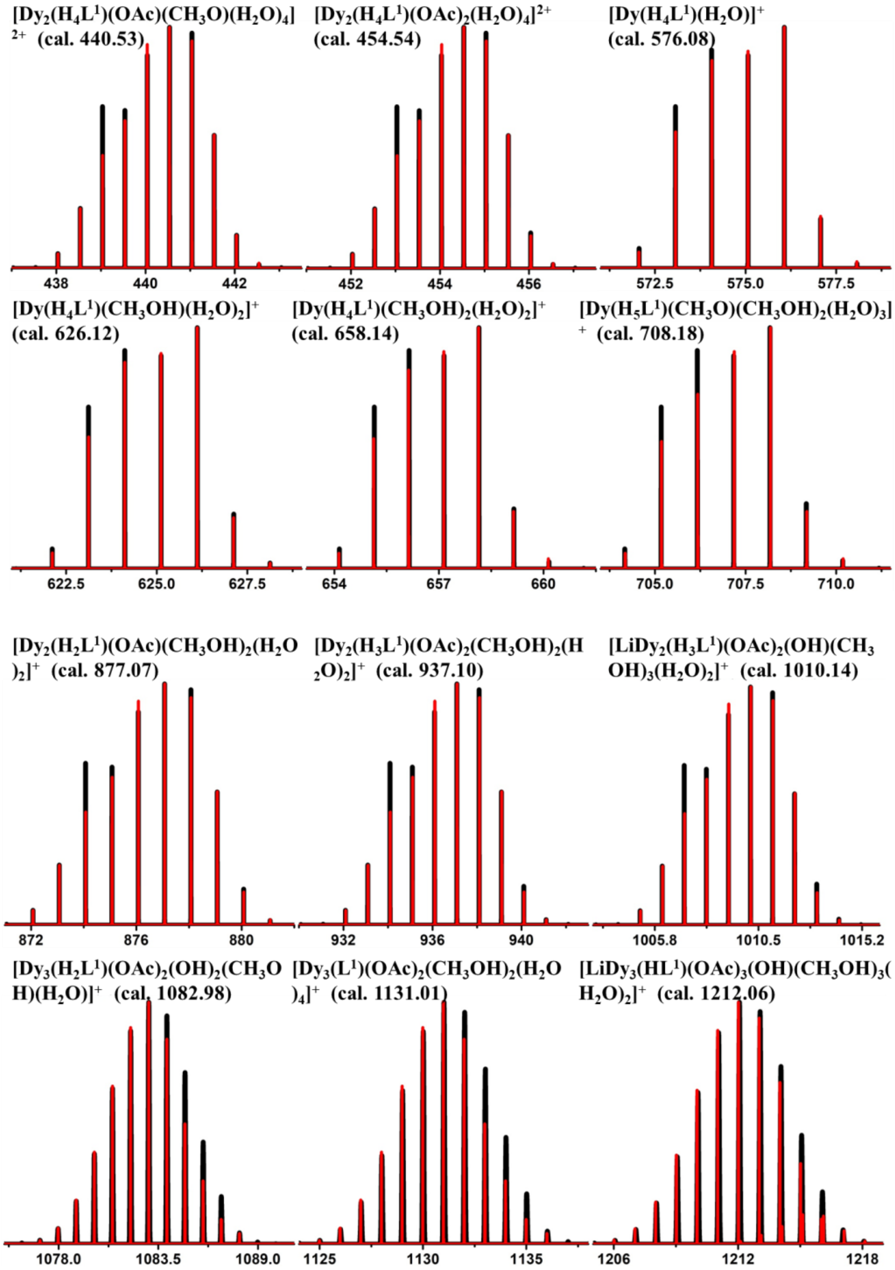

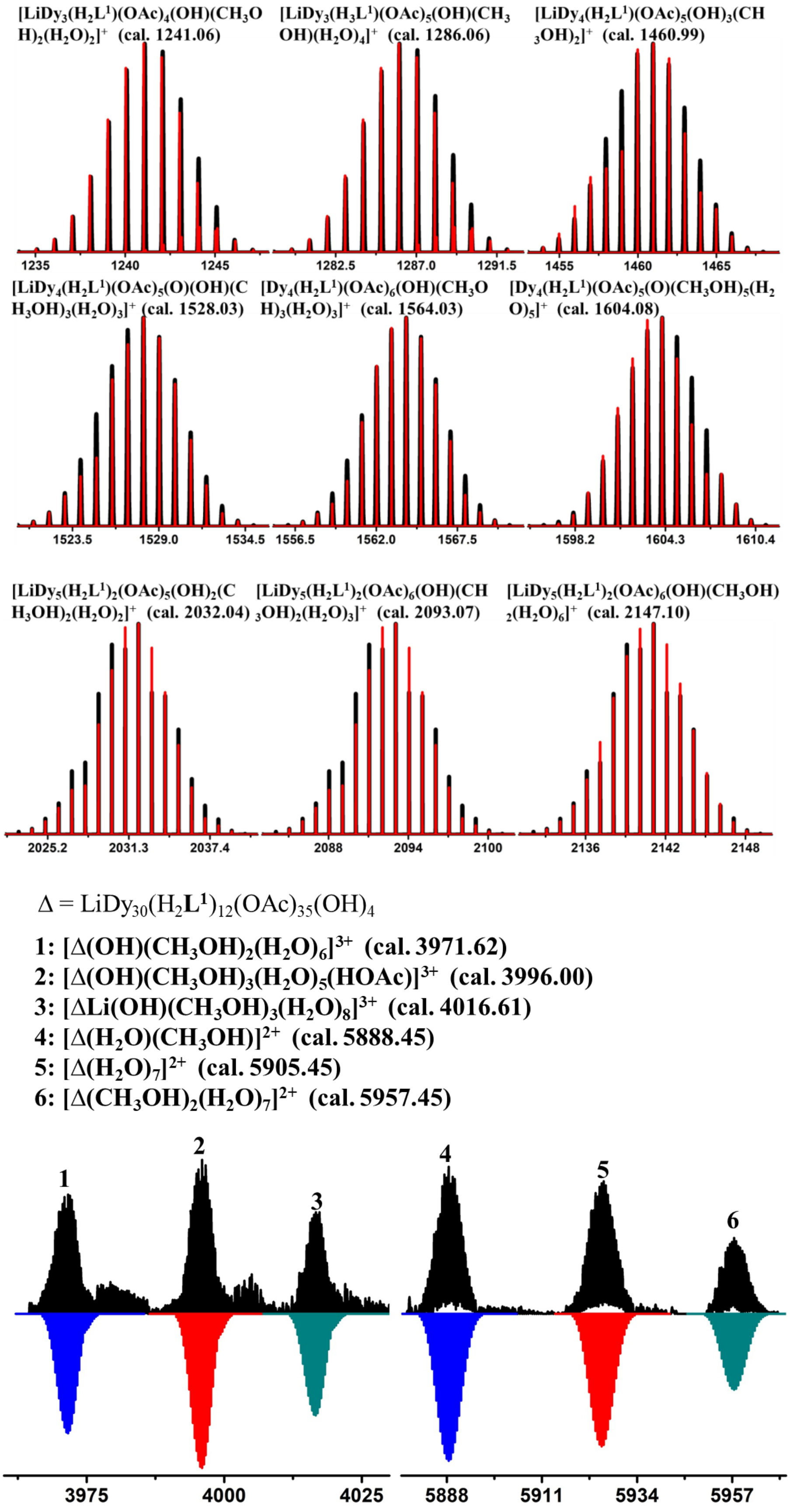

Supplementary Figure 11 The superposed simulated and observed spectra of several species in the time-dependent HRESI-MS of **Dy_{30}** (positive mode).

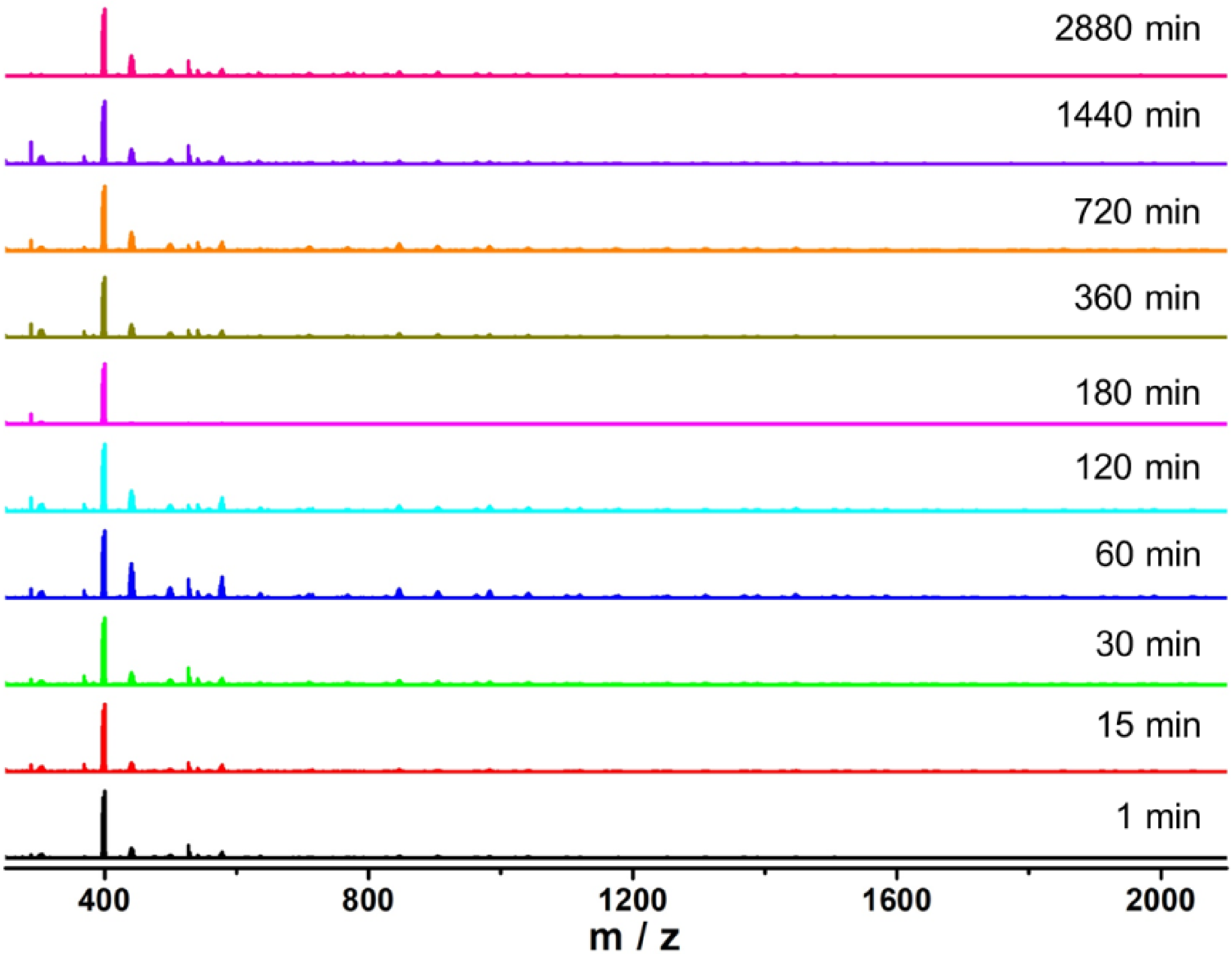

Supplementary Figure 12 Time-dependent HRESI-MS spectra of $\mathbf{Dy_{30}}$ in negative mode.

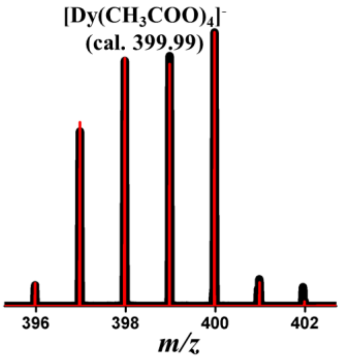

Supplementary Figure 13 The superposed simulated and observed spectra of several species in the time-dependent HRESI-MS of $\mathbf{Dy_{30}}$ (negative mode).

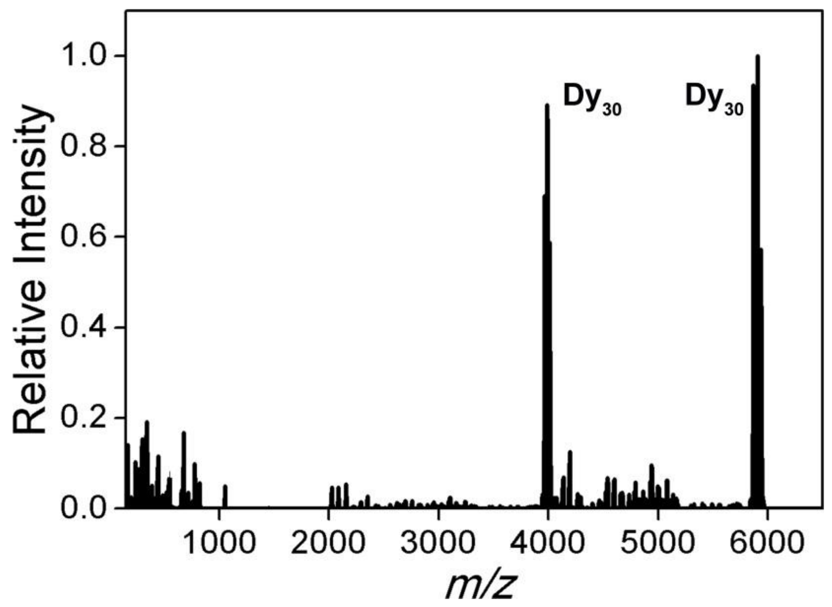

Supplementary Figure 14 Cationic HRESI-MS spectra of $\mathbf{Dy_{30}}$.

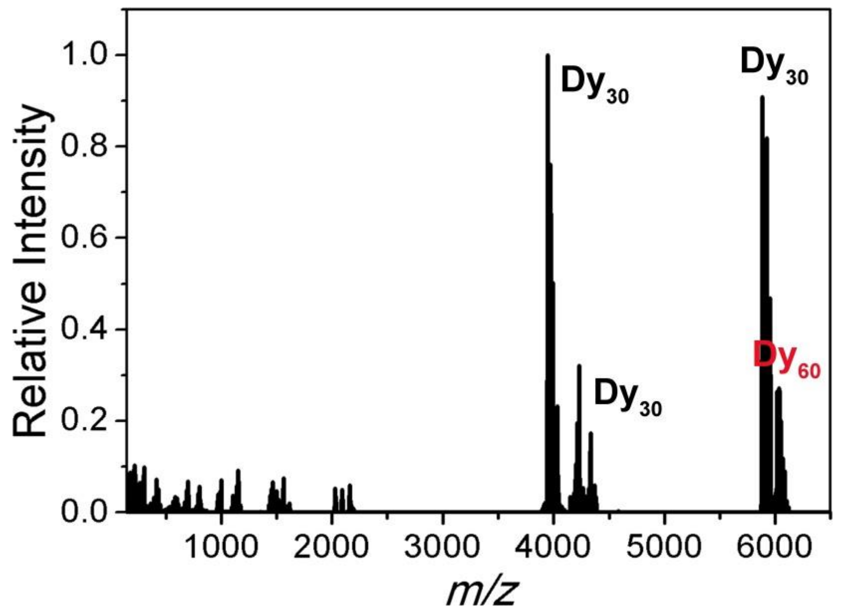

Supplementary Figure 15 Cationic HRESI-MS spectra of $\mathbf{Dy_{60}}$.

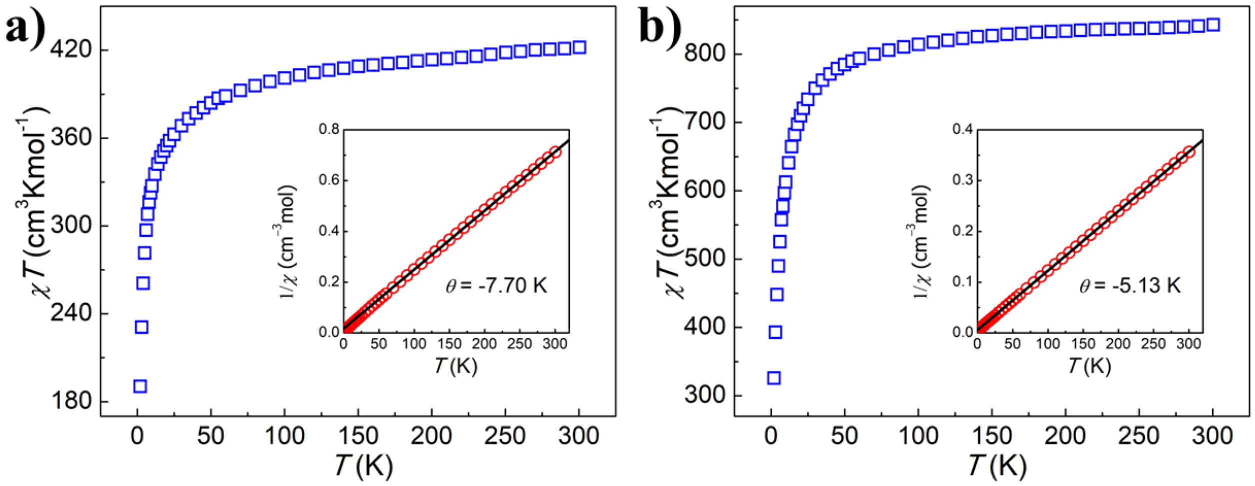

Supplementary Figure 16 Temperature dependence of $\chi_m T$ for $\mathbf{Dy_{30}}$ (a) and $\mathbf{Dy_{60}}$ (b).

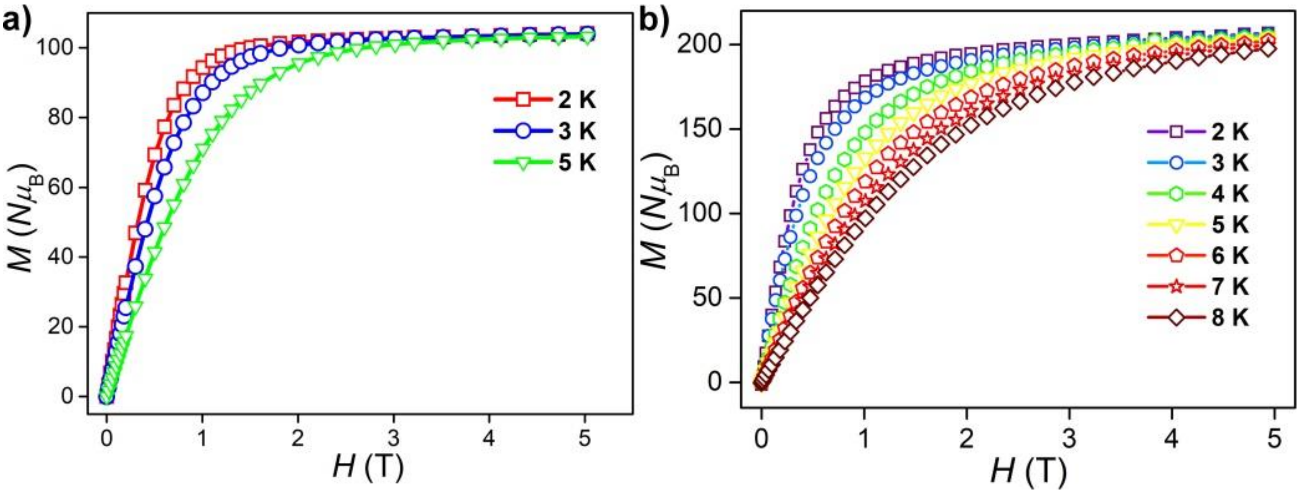

Supplementary Figure 17 *M* vs. *H* plots for $\mathbf{Dy_{30}}$ (a) and $\mathbf{Dy_{60}}$ (b).

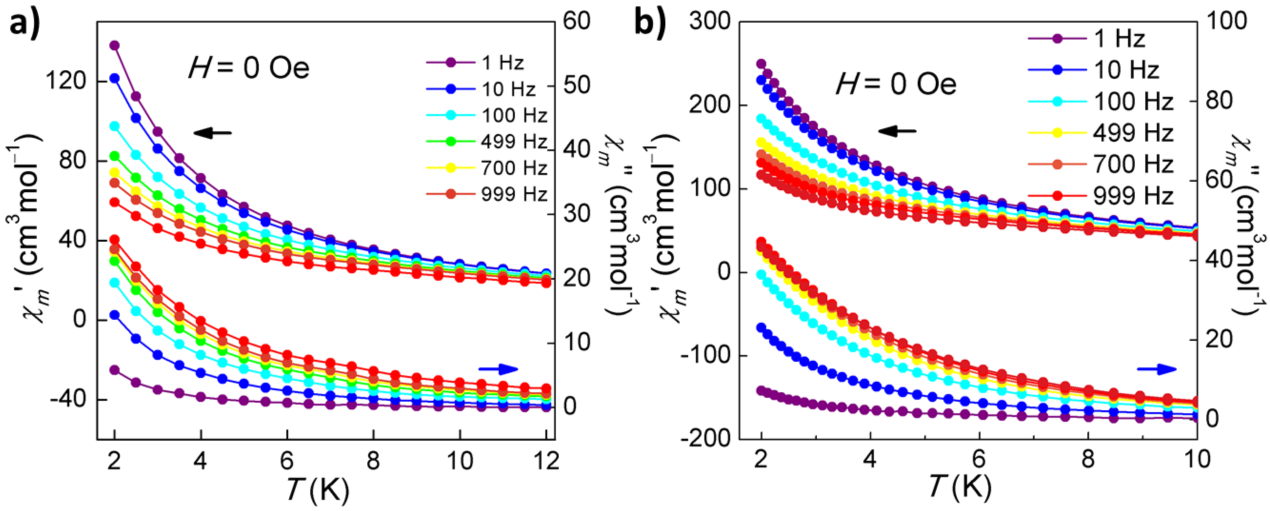

Supplementary Figure 18 Temperature-dependent in-phase (χ') and out-of phase (χ'') ac susceptibilities under 0 Oe dc field for for $\mathbf{Dy_{30}}$ (a) and $\mathbf{Dy_{60}}$ (b).

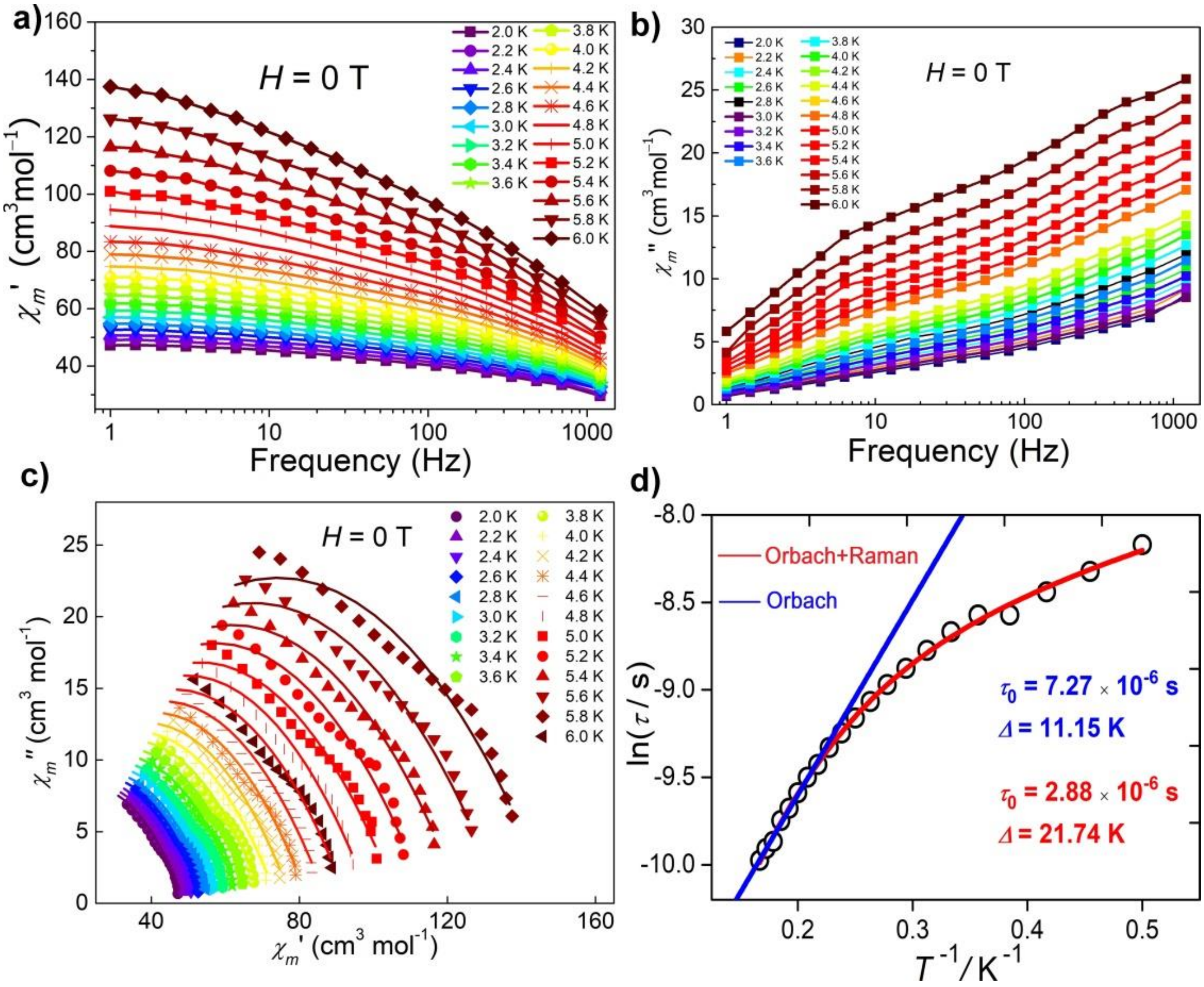

Supplementary Figure 19 Frequency-dependent in-phase (χ') and out-of phase (χ'') ac susceptibilities under 0 Oe dc fields for **Dy_{30}** (a and b) and Cole–Cole plots (c) under 0 Oe at different temperatures with the solid lines guiding for eyes and representing the best fitting, respectively. The ln(τ/s) versus T^{-1} curves of **Dy_{30}** (d) with the fit to the Arrhenius equation represented by solid lines.

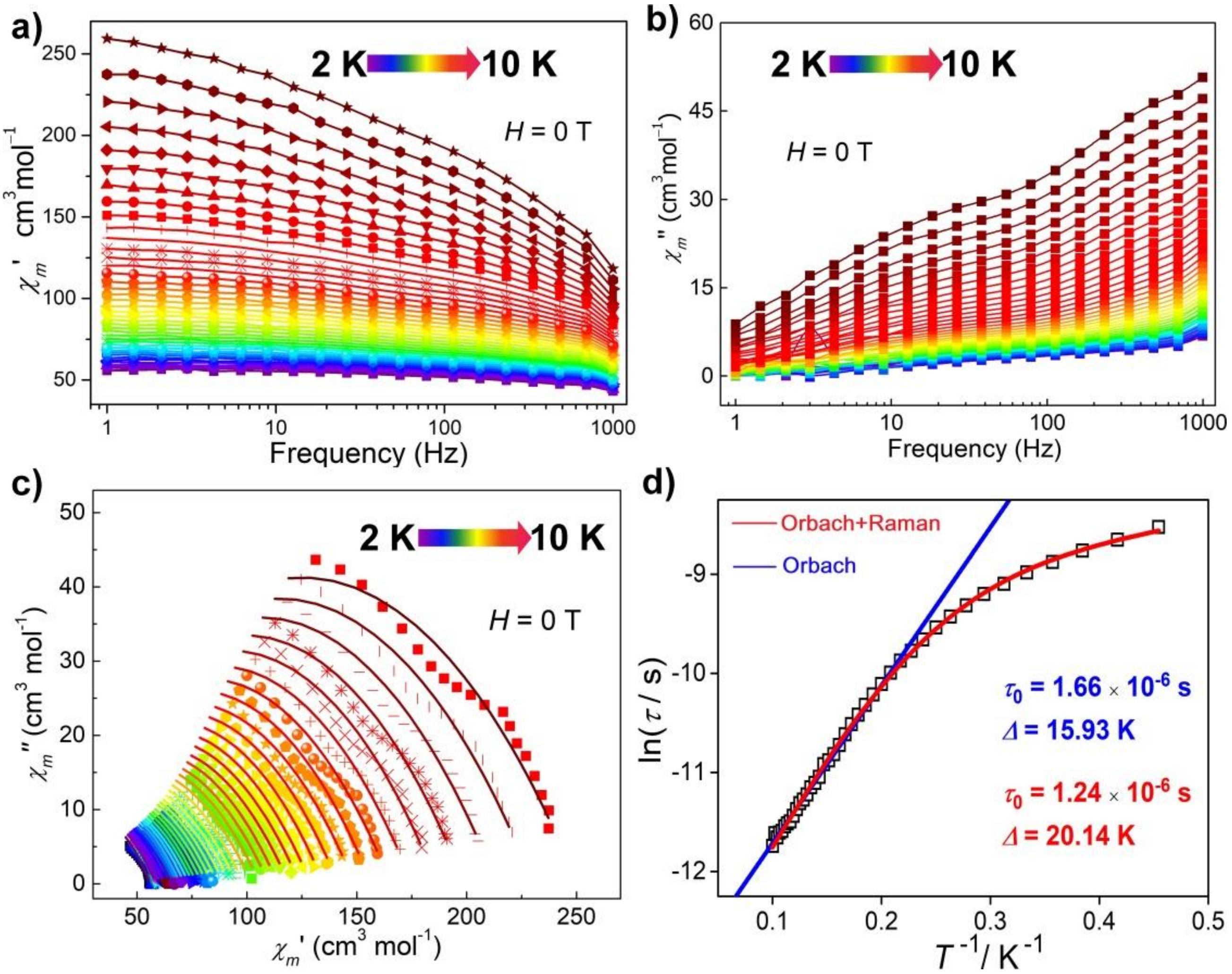

Supplementary Figure 20 Frequency-dependent in-phase (χ') and out-of phase (χ'') ac susceptibilities under 0 Oe dc fields for **Dy_{60}** (a and b) and Cole–Cole plots (c) under 0 Oe at different temperatures with the solid lines guiding for eyes and representing the best fitting, respectively. The ln(τ/s) versus T^{-1} curves of **Dy_{60}** (d) with the fit to the Arrhenius equation represented by solid lines.

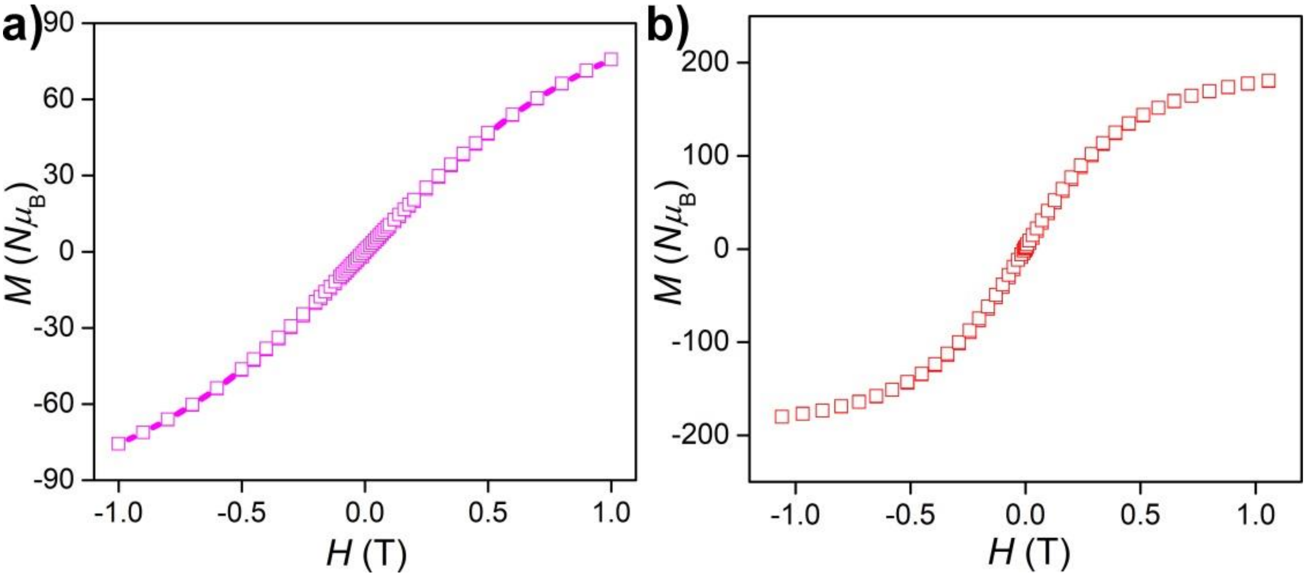

Supplementary Figure 21 Loop plots for **Dy$_{30}$** (a) and **Dy$_{60}$** (b).

Supplementary Table 1 43 examples of high-nuclear lanthanide clusters are known with nuclearity ≥ 10 was queried using Scifinder until 15 Oct. 2019. The number of genuine high-nuclear lanthanide clusters may be varied because of the term “high-nuclear lanthanide clusters” was not used in some papers.

No	Complex	Ref.
1	$[Ln_{14}(CO_3)_{13}(ccnm)_9(OH)(H_2O)_6(phen)_{13}(NO_3)]\cdot(CO_3)_{2.5}\cdot(phen)_{0.5}$ (**Ln$_{14}$**)	2
2	$[Ln_{24}(DMC)_{36}(\mu_4\text{-}CO_3)_{18}(\mu_3\text{-}H_2O)_2]$ (**Ln$_{24}$**)	3, 4
3	$[(CO_3)_2@Ln_{37}(LH_3)_8(CH_3COO)_{21}(CO_3)_{12}(\mu_3\text{-}OH)_{41}(\mu_2\text{-}H_2O)_5(H_2O)_{40}]\cdot(ClO_4)_{21}\cdot100(H_2O)$ (**Ln$_{37}$**)	5
4	$[Er_{60}(L\text{-}thre)_{34}(\mu_6\text{-}CO_3)_8(\mu_3\text{-}OH)_{96}(\mu_2\text{-}O)_2(H_2O)_{18}]\cdot Br_{12}\cdot(ClO_4)_{18}\cdot40(H_2O)$ (**Ln$_{60}$**)	6
5	$[Dy_{72}(mda)_{24}(mdaH)_8(OH)_{120}(O)_8(NO_3)_{16}]\cdot(NO_3)_8$ (**Ln$_{72}$**)	7
6	$[Gd_{38}(\mu\text{-}O)(\mu_8\text{-}ClO_4)_6(\mu_3\text{-}OH)_{42}(CAA)_{37}(H_2O)_{36}(EtOH)_6]\cdot(ClO_4)_{10}\cdot(OH)_{17}\cdot14DMSO\cdot13H_2O$ (**Ln$_{38}$**)	8
7	$[Gd_{48}(\mu_4\text{-}O)_6(\mu_3\text{-}OH)_{84}(CAA)_{36}(NO_3)_6(H_2O)_{24}(EtOH)_{12}(NO_3)Cl_2]\cdot Cl_3$ (**Ln$_{48}$**)	
8	$[Ln_{104}(ClO_4)_6(CH_3COO)_{56}(\mu_3\text{-}OH)_{168}(\mu_4\text{-}O)_{30}(H_2O)_{112}]\cdot(ClO_4)_{22}$ (**Ln$_{104}$**)	9
9	$\{[Ln_{36}(NA)_{36}(OH)_{49}(O)_6(NO_3)_6(N_3)_3(H_2O)_{20}]Cl_2\cdot28H_2O\}_n$ (**Ln$_{36}$**)	10
10	$\{[Cl_2\&(NO_3)]@[Er_{48}(NA)_{44}(OH)_{90}(N_3)(H_2O)_{24}]\}_n$ (**Ln$_{48}$**)	11
11	$K_2[Ho_{48}(IN)_{46}(\mu_3\text{-}OH)_{84}(\mu_4\text{-}OH)_4(\mu_5\text{-}O)_2(OAc)_4(H_2O)_{14}(CO_3)Br_2]$ (**Ln$_{48}$**)	12
12	$[(ClO_4)@Ln_{27}(\mu_3\text{-}OH)_{32}(CO_3)_8(CH_3CH_2COO)_{20}(H_2O)_{40}]\cdot(ClO_4)_{12}\cdot(H_2O)_{50}$ (**Ln$_{27}$**)	13
13	$[Ln_{15}(\mu_3\text{-}OH)_{20}(\mu_5\text{-}X)]^{24+}$ (**Ln$_{15}$**)	14, 15
14	$[Dy_{19}$(**1**-3H)(**1**-2H)$_{11}(CH_3CO_2)_6(OH)_{26}(H_2O)_{30}]$ (**Ln$_{19}$**)	16
15	$Ln_{14}(\mu_4\text{-}OH)_2(\mu_3\text{-}OH)_{16}(\mu\text{-}\eta^2\text{-}acac)_8(\eta^2\text{-}acac)_{16}$ (**Ln$_{14}$**)	17
16	$H_{18}[Ln_{14}(\mu\text{-}\eta2\text{-}O_2N\text{-}C_6H_4\text{-}O)_8(\eta2\text{-}O_2N\text{-}C_6H_4\text{-}O)_{16}(\mu_4\text{-}O)_2(\mu_3\text{-}O)_{16}]$ (**Ln$_{14}$**)	18
17	$Ln_{14}(\mu_4\text{-}OH)_2(\mu_3\text{-}OH)_{16}(\mu\text{-}\eta^2\text{-}acac)_8(\eta^2\text{-}acac)_{16}\cdot6H_2O$ (**Ln$_{14}$**)	19
18	$[Ho_{26}(IN)_{28}(CH_3COO)_4(CO_3)_{10}(OH)_{26}(H_2O)_{18}]\cdot20H_2O$ (**Ln$_{26}$**)	20
19	$[Dy_{26}(\mu_3\text{-}OH)_{20}(\mu_3\text{-}O)_6(NO_3)_9I]^{36+}$ (**Ln$_{26}$**)	21

20	$[Gd_{10}(\mu_3\text{-}OH)_8]^{22+}$ (**Ln_{10}**)	22
21	$[Dy_{10}O_2(OH)_6(o\text{-}van)_6(ISO)_{13}(H_2O)_2](NO_3)$ (**Ln_{10}**)	23
22	$[Ln_{10}(TBC8A)_2(PhPO_3)_4(OH)_2(HCO_3)(HCOO)(DMF)_{14}]\cdot(H_6TBC8A)\cdot 8CH_3OH$ (**Ln_{10}**)	24
23	$[Ln_{16}As_{16}W_{164}O_{576}(OH)_8(H_2O)_{42}]^{80-}$ (**Ln_{16}**)	25
24	$[Ln_{27}Ge_{10}W_{106}O_{406}(OH)_4(H_2O)_{24}]^{59-}$ (**Ln_{26}**)	26
25	$[Ln_{12}(L)_6(OH)_4O_2(CO_3)_6][Ln_{12}(L)_6(OH)_4O_4(CO_3)_6]\cdot(ClO_4)_4\cdot xH_2O$ (**Ln_{12}**)	27
26	$[Dy_{11}(OH)_{11}(phendox)_6(phenda)_3(OAc)_3]\cdot(OH)\cdot 40H_2O\cdot 7MeOH$ (**Ln_{11}**)	28
27	$[Gd_{60}(CO_3)_8(CH_3COO)_{12}(\mu_2\text{-}OH)_{24}(\mu_3\text{-}OH)_{96}(H_2O)_{56}](NO_3)_{15}\cdot Br_{12}\cdot(dmp)_5\cdot 30CH_3OH\cdot 20Hdmp$ (**Ln_{60}**)	29
28	$[Ln_{12}(fsa)_{12}(\mu f_3\text{-}OH)_{12}(DMF)_{12}]\cdot n DMF$ (**Ln_{12}**)	30
29	$(Pr_2NH_2)_5[Dy_{12}(OH)_{16}(SALO)_4(SALOH)_8(NO_3)_8(H_2O)_{0.5}]NO_3$ (**Ln_{12}**)	31
30	$[Dy_{12}(L)_8(OH)_{16}(CH_3O)_8(H_2O)_8]\cdot(CH_3O)_4$ (**Ln_{12}**)	32
31	$(H_3O)_6[Dy_{76}O_{10}(OH)_{138}(OAc)_{20}(L_1)_{44}(H_2O)_{34}]\cdot 2CO_3\cdot 4Cl\cdot 2L_1\cdot 2OAc$ (**Dy_{76}**)	33
32	$[Gd_{18}(ovpho)_6(OAc)_{30}(H2O)_6]\cdot 21CH_3OH\cdot 18H_2O$ (**Gd_{18}**)	34
33	$[Ln_{11}(ovpho)_4(\mu\text{-}CH_3O)_2(\mu\text{-}H_2O)_2(\mu_3\text{-}OH)_6(CH_3OH)_4(H_2O)_2(NO_3)_8](OH)\cdot xH_2O\cdot yCH_3OH$ (**Ln_{11}**)	35
34	$[Ln(\mu_3\text{-}OH)_8][Ln_{16}(\mu_4\text{-}O)(\mu_4\text{-}OH)(\mu_3\text{-}OH)_8(H_2O)_8(\mu_4\text{-}dcd)_8][(\mu_3\text{-}dcd)_8]\cdot 22H_2O$ (**Ln_{17}**)	36
35	$[Dy_{21}(L)_7(LH)_7(tfa)_7]\cdot Cl_7\cdot 15H_2O\cdot 7MeOH\cdot 12CHCl_3$ (**Ln_{21}**)	37
36	$Dy_{10}(MOE)_{30}$ (**Dy_{10}**)	38
37	$[Dy_{10}(\mu_3\text{-}OH)_4(OAc)_{20}(H_2L)_2(H_3L)_2\{NH_2C(CH_2OH)_3\}_2]$ (**Dy_{10}**)	39
38	$\{Dy_{12}(OH)_{16}(phenda)_8(H_2O)_8\}]^{2+}$ (**Dy_{12}**)	40
39	$[Dy_{14}(EDDC)_4(opch)_4(O_3PC_{10}H_7)_{10}(OAc)_6(H_2O)_4]\cdot xH_2O$ (**Dy_{14}**)	41
40	$[\{Dy_{15}(OH)_{20}(PepCO_2)_{10}(DBM)_{10}Cl\}Cl_4]$ (**Dy_{15}**)	42
41	$[Et_4N]_3[Ln_{16}(\mu_3\text{-}OH)_{24}(bsc)_6(H_2O)_{18}]Cl_{15}\cdot 2H_2O\cdot EtOH$ (**Dy_{16}**)	43
42	$[Dy_{20}(\mu_4\text{-}O)_{11}(\mu_3\text{-}OMe)_{12}(\mu\text{-}OMe)_8(MebptpO)_4(PhCO_2)_8(H_2O)_4](OH)_2\cdot xH_2O\cdot yMeOH\cdot zMeCN$ (**Dy_{20}**)	44
43	$\{Dy_5(EDDC)_2(\mu_3\text{-}AcO)_2(\mu_5\text{-}C_{15}H_{11}PO_3)(\mu_4\text{-}C_{15}H_{11}PO_3)(\mu_2\text{-}AcO)_2(AcO)_2(H_2O)(CH_3OH)_2\}_2(\mu_4\text{-}C_2O_4)\cdot xH_2O$ (**Dy_{10}**)	45

Supplementary Table 2 Crystallographic data of the complexes **Dy_{30}** and **Dy_{60}**.

**Complexes**	**Dy_{30}**	**Dy_{60}**
Formula	$C_{338}H_{389}Dy_{30}N_{85}O_{172}$	$C_{618}H_{615}Dy_{60}N_{151}O_{309}$
Formula weight	13269.17	24851.33
T (K)	153	293(2)
Crystal system	Trigonal	Triclinic
Space group	*P*-3*c*1	*P*-1
a (Å)	36.51215(17)	22.0753(2)
b (Å)	36.51215(17)	43.9055(4)

c (Å)	50.7414(2)	46.8047(3)
α (°)	90	86.7860(10)
β (°)	90	84.755(2)
γ (°)	120	79.0290(10)
V ($Å^3$)	58582.5(5)	44314.1(7)
Z	4	2
D_c(g cm^{-3})	1.380	1.818
μ (mm^{-1})	20.60	27.221
Reflns coll.	371911	473255
Unique reflns	40315	164023
R_{int}	0.064	0.0109
$^aR_1[I \geq 2\sigma(I)]$	0.040	0.0332
$^bwR_2$(all data)	0.120	0.0699
GOF	1.076	1.051

$^aR_1 = \Sigma||F_o|-|F_c||/\Sigma|F_o|$, $^bwR_2 = [\Sigma w(F_o^2-F_c^2)^2/\Sigma w(F_o^2)^2]^{1/2}$

Supplementary Table 3 Major species assigned in the Time-dependent HRESI-MS of $\mathbf{Dy_{60}}$ in positive mode.

m/z	Fragment	Relative Intensity						
		0min	30min	1 h	2 h	3 h	12 h	48 h
447.54	$[Dy_2(H_4\mathbf{L}^1)(OAc)(CH_3O)(CH_3OH)(H_2O)_3]^{2+}$ (cal. 447.54)	0	0.093	0.278	0.529	0.402	0.084	0
468.54	$[Dy_2(H_4\mathbf{L}^1)(OAc)_2(CH_3OH)_2(H_2O)_2]^{2+}$ (cal. 454.54)	0.103	0.663	1	0.684	0.105	0	0
491.56	$[Dy_2(H_4\mathbf{L}^1)(OAc)_2(CH_3OH)_2(H_2O)_2]^{2+}$ (cal. 491.56)	0.004	0.893	0.952	0.703	0.385	0	0
558.07	$[Dy(H_4\mathbf{L}^1)]^+$ (cal. 558.07)	0.378	0.897	0.632	0.279	0.303	0	0
622.12	$[Dy(H_4\mathbf{L}^1)(CH_3OH)_2]^+$ (cal. 622.12)	0.298	0.922	0.721	0.219	0.005	0	0
663.15	$[Dy(H_4\mathbf{L}^1)(CH_3OH)_2(CH_3CN)]^+$ (cal. 663.15)	1	1	0.853	0.404	0.172	0	0
704.17	$[Dy(H_5\mathbf{L}^1)(CH_3O)(CH_3OH)_2(CH_3CN)_2]^+$ (cal. 704.17)	0.901	0.863	0.583	0.117	0.008	0	0
798.53	$[Dy_4(H_2\mathbf{L}^1)(OAc)_6(CH_3OH)_4(H_2O)_4]^{2+}$ (cal. 799.53)	0	0.287	0.409	0.749	0.905	0.287	0
882.07	$[Dy_2(H_2\mathbf{L}^1)(OAc)(CH_3OH)_2(CH_3CN)]^+$	0.092	0.729	0.964	0.904	0.617	0.005	0

	(cal. 882.07)							
942.09	$[Dy_2(H_3\mathbf{L}^{\mathbf{1}})(OAc)_2(CH_3OH)_2(CH_3CN)]^+$ (cal. 942.09)	0.086	0.582	0.806	0.722	0.471	0	0
983.12	$[Dy_2(H_3\mathbf{L}^{\mathbf{1}})(OAc)_2(CH_3OH)_2(CH_3CN)_2]^+$ (cal. 983.12)	0.041	0.513	0.767	0.710	0.385	0	0
1085.98	$[Dy_3(H_2\mathbf{L}^{\mathbf{1}})(OAc)_2(OH)_2(H_2O)_3]^+$ (cal. 1085.97)	0.064	0.484	0.853	1	0.683	0.017	0
1133.03	$[Dy_3(\mathbf{L}^{\mathbf{1}})(OAc)_2(CH_3OH)_3(CH_3CN)]^+$ (cal. 1133.03)	0.082	0.408	0.795	0.901	0.699	0.027	0
1275.08	$[Dy_3(H\mathbf{L}^{\mathbf{1}})(OAc)_3(CH_3OH)_3(CH_3CN)_3]^+$ (cal. 1275.10)	0.018	0.383	0.738	0.885	0.589	0.052	0
1362.15	$[Dy_3(H_2\mathbf{L}^{\mathbf{1}})(OAc)_3(CH_3OH)_4(H_2O)_3(CH_3CN)_3]^+$ (cal. 1362.16)	0.073	0.401	0.722	0.900	0.716	0.094	0
1457.96	$[Dy_4(H_2\mathbf{L}^{\mathbf{1}})(OAc)_5(O)(H_2O)_4(CH_3OH)]^+$ (cal. 1457.97)	0.001	0.183	0.581	0.884	1	0.299	0
1513.01	$[Dy_4(H_2\mathbf{L}^{\mathbf{1}})(OAc)_5(O)(CH_3CN)(CH_3OH)_2(H_2O)_3]^+$ (cal. 1513.01)	0.002	0.216	0.538	0.826	0.898	0.238	0
1564.02	$[Dy_4(H_2\mathbf{L}^{\mathbf{1}})(OAc)_6(OH)(CH_3OH)_3(H_2O)_3]^+$ (cal. 1564.03)	0	0.173	0.474	0.762	0.798	0.187	0
1613.08	$[Dy_4(H_2L)(OAc)_5(O)(CH_3OH)_4(CH_3CN)(H_2O)_5]^+$ (cal. 1613.08)	0	0.152	0.430	0.803	0.728	0.201	0
2031.03	$[Dy_5(H_2\mathbf{L}^{\mathbf{1}})_2(OAc)_5(OH)(CH_3OH)(H_2O)_5]^+$ (cal. 2031.03)	0	0.093	0.285	0.716	0.906	0.722	0.009
2092.06	$[Dy_5(H_2\mathbf{L}^{\mathbf{1}})_2(OAc)_6(CH_3OH)(H_2O)_6]^+$ (cal. 2092.06)	0	0.068	0.239	0.757	0.884	0.663	0
2166.10	$[Dy_5(H_4\mathbf{L}^{\mathbf{1}})_2(OAc)_6(CH_3OH)_2(H_2O)_8]^+$ (cal. 2166.10)	0	0.079	0.264	0.698	0.897	0.603	0.007
3944.29	$[Dy_{30}(H_2\mathbf{L}^{\mathbf{1}})_{12}(OAc)_{35}(OH)_4(H_2O)(CH_3OH)_3]^{3+}$ (cal.3944.30)	0	0	0.002	0.146	0.327	0.761	0.761
3968.64	$[Dy_{30}(H_2\mathbf{L}^{\mathbf{1}})_{12}(OAc)_{35}(OH)_4(H_2O)_5(CH_3OH)_3]^{3+}$ (cal.3968.63)	0	0	0.003	0.192	0.429	1	1
3989.99	$[Dy_{30}(H_2\mathbf{L}^{\mathbf{1}})_{12}(OAc)_{35}(OH)_4(H_2O)_7(CH_3OH)_4]^{3+}$ (cal.3990.00)	0	0	0.001	0.097	0.210	0.502	0.501
5884.44	$[Dy_{30}(H_2\mathbf{L}^{\mathbf{1}})_{12}(OAc)_{35}(OH)_5(H_2O)_2]^{2+}$ (cal.5884.42)	0	0	0.002	0.160	0.355	0.840	0.867
5921.95	$[Dy_{30}(H_2\mathbf{L}^{\mathbf{1}})_{12}(OAc)_{35}(OH)_5(H_2O)_6]^{2+}$ (cal.5925.45)	0	0	0.002	0.178	0.394	0.932	0.962
5953.46	$[Dy_{30}(H_2\mathbf{L}^{\mathbf{1}})_{12}(OAc)_{35}(OH)_5(H_2O)_6(CH_3OH)_2]^{2+}$ (cal.5953.45)	0	0	0.001	0.091	0.203	0.480	0.496
6017.50	$[Dy_{60}(H_2\mathbf{L}^{\mathbf{1}})_{24}(OAc)_{71}(OH)_{10}(H_2O)_{16}(CH_3OH)_6(HOAc)]^{4+}$ (cal.6017.50)	0	0	0	0	0.096	0.213	0.227
6037.02	$[Dy_{60}(H_2\mathbf{L}^{\mathbf{1}})_{24}(OAc)_{71}(OH)_{10}(H_2O)_{24}(CH_3OH)_4(HOAc)]^{4+}$ (cal.6037.00)	0	0	0	0	0.099	0.218	0.233

6051.53	$[Dy_{60}(H_2\mathbf{L}^1)_{24}(OAc)_{71}(OH)_{10}(H_2O)_{20}(CH_3OH)_8(HOAc)]^{4+}$ (cal.6051.50)	0	0	0	0	0.073	0.161	0.172
6071.05	$[Dy_{60}(H_2\mathbf{L}^1)_{24}(OAc)_{71}(OH)_{10}(H_2O)_{28}(CH_3OH)_6(HOAc)]^{4+}$ (cal.6071.05)	0		0	0	0.043	0.095	0.102
6085.54	$[Dy_{60}(H_2\mathbf{L}^1)_{24}(OAc)_{71}(OH)_{10}(H_2O)_{24}(CH_3OH)_{10}(HOAc)]^{4+}$(cal.6085.55)	0	0	0	0	0.033	0.073	0.078

Supplementary Table 4 Major species assigned in the time-dependent HRESI-MS of $\mathbf{Dy_{30}}$ in positive mode.

m/z	Fragment	Relative Intensity						
		0min	30min	1 h	2 h	3 h	12 h	48 h
440.54	$[Dy_2(H_4\mathbf{L}^1)(OAc)(CH_3O)(H_2O)_4]^{2+}$ (cal. 440.53)	0.127	0.739	0.915	0.628	0.282	0	0
454.53	$[Dy_2(H_4\mathbf{L}^1)(OAc)_2(H_2O)_4]^{2+}$ (cal. 454.54)	0.119	0.656	0.852	0.743	0.302	0	0
576.07	$[Dy(H_4\mathbf{L}^1)(H_2O)]^+$ (cal. 576.08)	1	0.905	0.789	0.547	0.272	0	0
626.12	$[Dy(H_4\mathbf{L}^1)(CH_3OH)(H_2O)_2]^+$ (cal. 626.12)	0.973	0.965	0.752	0.496	0.201	0	0
658.15	$[Dy(H_4\mathbf{L}^1)(CH_3OH)_2(H_2O)_2]^+$ (cal. 658.14)	0.952	0.895	0.717	0.540	0.237	0.001	0
708.17	$[Dy(H_5\mathbf{L}^1)(CH_3O)(CH_3OH)_2(H_2O)_3]^+$ (cal. 708.18)	0.874	0.876	0.639	0.508	0.316	0.002	0
877.07	$[Dy_2(H_2\mathbf{L}^1)(OAc)(CH_3OH)_2(H_2O)_2]^+$ (cal. 877.07)	0.163	0.795	0.897	0.698	0.415	0	0
937.09	$[Dy_2(H_3\mathbf{L}^1)(OAc)_2(CH_3OH)_2(H_2O)_2]^+$ (cal. 937.10)	0.284	1	0.905	0.532	0.183	0	0
1010.14	$[LiDy_2(H_3\mathbf{L}^1)(OAc)_2(OH)(CH_3OH)_3(H_2O)_2]^+$ (cal. 1010.14)	0.116	0.803	0.785	0.499	0.176	0	0
1082.98	$[Dy_3(H_2\mathbf{L}^1)(OAc)_2(OH)_2(CH_3OH)(H_2O)]^+$ (cal. 1082.98)	0	0.574	1	0.926	0.635	0.002	0
1131.02	$[Dy_3(\mathbf{L}^1)(OAc)_2(CH_3OH)_2(H_2O)_4]^+$ (cal. 1131.01)	0	0.529	0.864	0.941	0.597	0.005	0
1212.06	$[LiDy_3(H\mathbf{L}^1)(OAc)_3(OH)(CH_3OH)_3(H_2O)_2]^+$ (cal. 1212.06)	0	0.473	0.806	0.861	0.784	0.078	0
1241.07	$[LiDy_3(H_2\mathbf{L}^1)(OAc)_4(OH)(CH_3OH)_2(H_2O)_2]^+$ (cal. 1241.06)	0	0.394	0.683	0.510	0.328	0	0
1286.05	$[LiDy_3(H_3\mathbf{L}^1)(OAc)_5(OH)(CH_3OH)(H_2O)_4]^+$ (cal. 1286.06)	0.003	0.355	0.687	0.725	0.462	0	0
1460.98	$[LiDy_4(H_2\mathbf{L}^1)(OAc)_5(OH)_3(CH_3OH)_2]^+$ (cal. 1460.99)	0	0.381	0.764	1	0.861	0.188	0
1528.02	$[LiDy_4(H_2\mathbf{L}^1)(OAc)_5(O)(OH)(CH_3OH)_3(H_2O)_3]^+$ (cal. 1528.03)	0	0.279	0.654	0.903	0.748	0	0

1564.02	$[Dy_4(H_2\mathbf{L}^1)(OAc)_6(OH)(CH_3OH)_3(H_2O)_3]^+$ (cal. 1564.03)	0	0.252	0.476	0.751	0.594	0.012	0
1604.08	$[Dy_4(H_2L)(OAc)_5(O)(CH_3OH)_5(H_2O)_5]^+$ (cal. 1604.08)	0	0.193	0.374	0.520	0.481	0.108	0
2032.04	$[LiDy_5(H_2\mathbf{L}^1)_2(OAc)_5(OH)_2(CH_3OH)_2(H_2O)_2]^+$ (cal. 2032.04)	0	0.148	0.588	0.895	1	0.698	0.051
2093.07	$[LiDy_5(H_2\mathbf{L}^1)_2(OAc)_6(OH)(CH_3OH)_2(H_2O)_3]^+$ (cal. 2093.07)	0	0.127	0.489	0.518	0.727	0.415	0.017
2147.10	$[LiDy_5(H_2\mathbf{L}^1)_2(OAc)_6(OH)(CH_3OH)_2(H_2O)_6]^+$ (cal. 2147.10)	0	0.107	0.388	0.492	0.654	0.584	0.003
3971.63	$[LiDy_{30}(H_2\mathbf{L}^1)_{12}(OAc)_{35}(OH)_5(H_2O)_6(CH_3OH)_2]^{3+}$ (cal.3971.62)	0	0	0	0.076	0.212	0.723	0.700
3995.98	$[LiDy_{30}(H_2\mathbf{L}^1)_{12}(OAc)_{35}(OH)_5(H_2O)_5(HOAc)(CH_3OH)_3]^{3+}$ (cal.3996.00)	0	0	0	0.098	0.274	0.933	0.904
4016.63	$[Li_2Dy_{30}(H_2\mathbf{L}^1)_{12}(OAc)_{36}(OH)_5(H_2O)_8(CH_3OH)_3]^{3+}$ (cal.4016.61)	0	0	0	0.065	0.181	0.615	0.607
5888.44	$[LiDy_{30}(H_2\mathbf{L}^1)_{12}(CH3COO)_{35}(OH)_4(H_2O)(CH_3OH)]^{2+}$ (cal.5888.45)	0	0	0.001	0.127	0.437	0.935	0.921
5905.46	$[LiDy_{30}(H_2\mathbf{L}^1)_{12}(CH3COO)_{35}(OH)_4(H_2O)_7]^{2+}$ (cal.5905.45)	0	0	0	0.136	0.467	1	1
5957.46	$[LiDy_{30}(H_2\mathbf{L}^1)_{12}(CH3COO)_{35}(OH)_4(H_2O)_7(CH_3OH)_2]^{2+}$ (cal.5957.45)	0	0	0	0.078	0.267	0.572	0.613

Supplementary Table 5 ICP result of $\mathbf{Dy_{30}}$.

Element	Content / (μg / μg)				Percentage by weight/%			
	1	2	3	average	1	2	3	average
Dy	98.460	66.228	75.001	79.896	38.76	37.21	39.68	38.55

Supplementary Table 6 ICP result of $\mathbf{Dy_{60}}$.

Element	Content / (μg / μg)				Percentage by weight/%			
	1	2	3	average	1	2	3	average
Dy	74.932	83.414	66.907	75.084	32.72	35.64	35.97	34.77

Supplementary References

[1] Liu, M. et al. Calixarene-Based Nanoscale Coordination Cages. *Angew. Chem. Int. Ed.* **51**, 1585–1588 (2012).

[2] Chesman, A. S. R. et al. Tetradecanuclear polycarbonatolanthanoid clusters: Diverse coordination modes of carbonate providing access to novel core geometries. *Dalton Trans.* **41**, 10903–10909 (2012).

[3] Chang, L.-X. et al. A 24-Gd nanocapsule with a large magnetocaloric effect. *Chem. Commun.* **49**,

1055-1057 (2013).
[4] Li, W.; Xiong, G. Elongated Wells–Dawson type 24-nuclear lanthanide clusters: Luminescence and magnetic properties. *Inorg. Chem. Commun*. **59**, 1–4 (2015).
[5] Zhou, Y. et al. Three Giant Lanthanide Clusters Ln_{37} (Ln = Gd, Tb, and Eu) Featuring A Double-Cage Structure. *Inorg. Chem*. **56**, 2037–2041 (2017).
[6] Kong, X.-J. et al. A Chiral 60-Metal Sodalite Cage Featuring 24 Vertex-Sharing $[Er_4(\mu_3\text{-}OH)_4]$ Cubanes. *J. Am. Chem. Soc*. **131**, 6918–6919 (2009).
[7] Qin, L. et al. A "Molecular Water Pipe": A Giant Tubular Cluster $\{Dy_{72}\}$ Exhibits Fast Proton Transport and Slow Magnetic Relaxation. *Adv. Mater.* **28**,10772-10779 (2016).
[8] Guo, F.-S. et al. Anion-Templated Assembly and Magnetocaloric Properties of a Nanoscale $\{Gd_{38}\}$ Cage versus a $\{Gd_{48}\}$ Barrel. *Chem. Eur. J*. **19**, 14876–14885 (2013).
[9] Peng, J.-B. et al. Beauty, Symmetry, and Magnetocaloric Effect-Four-Shell Keplerates with 104 Lanthanide Atoms. *J. Am. Chem. Soc*. **136**, 17938–17941 (2014).
[10] Wu, M. et al. Two polymeric 36-metal pure lanthanide nanosize clusters. *Chem. Sci*. **4**, 3104–3109 (2013).
[11] Wu, M. et al. Polymeric double-anion templated Er_{48} nanotubes. *Chem. Commun*. **50**, 1113–1115 (2014).
[12] Chen, L. et al. A novel 2-D coordination polymer constructed from high-nuclearity waist drum-like pure Ho_{48} clusters. *Chem. Commun*. **49**, 9728–9730 (2013).
[13] Zheng, X.-Y. et al. Mixed-anion templated cage-like lanthanide clusters: Gd_{27} and Dy_{27}. *Inorg. Chem. Front.* **3**, 320–325 (2016).
[14] Wang, R.; Zheng, Z.; Jin, T.; Staples, R. J. Coordination Chemistry of Lanthanides at "High" pH: Synthesis and Structure of the Pentadecanuclear Complex of Europium(III) with Tyrosine. *Angew. Chem. Int. Ed*. **38**, 1813–1815 (1999).
[15] Wang, R. et al. Halide-Templated Assembly of Polynuclear Lanthanide-Hydroxo Complexes. *Inorg. Chem.* **41**, 278−286 (2002).
[16] D'Alessio, D. et al. Lanthanoid "Bottlebrush" Clusters: Remarkably Elongated Metal−Oxo Core Structures with Controllable Lengths. *J. Am. Chem. Soc.* **136**, 15122−15125 (2014).
[17] Wang, R.; Song, D.; Wang, S. Toward constructing nanoscale hydroxo–lanthanide clusters: syntheses and characterizations of novel tetradecanuclear hydroxo–lanthanide clusters. *Chem. Commun*. 368–369 (2002).
[18] Bürgstein, M. R.; Gamer, M. T.; Roesky, P. W. Nitrophenolate as a Building Block for Lanthanide Chains, Layers, and Clusters. *J. Am. Chem. Soc.* **126**, 5213-5218 (2004).
[19] Li, X.-L. et al. Two chiral tetradecanuclear hydroxo-lanthanide clusters with luminescent and magnetic properties. *CrystEngComm.* **13**, 3643–3645 (2011).
[20] Chen, L. et al. Hydrothermal synthesis, structure, and properties of two new nanosized Ln_{26} (Ln = Ho, Er) clusters. *J. Coord. Chem*. **65**, 958–968 (2012).
[21] Gu, X.; Xue, D. Surface Modification of High-Nuclearity Lanthanide Clusters: Two Tetramers Constructed by Cage-Shaped $\{Dy_{26}\}$ Clusters and Isonicotinate Linkers. *Inorg. Chem.* **46**, 3212–3216 (2007).
[22] Liu, S. J. et al. An Unprecedented Decanuclear Gd^{III} Cluster for Magnetic Refrigeration. *Inorg. Chem*. **52**, 9163–9165 (2013).
[23] Langley, S. K. et al. Trinuclear, octanuclear and decanuclear dysprosium(III) complexes: Synthesis, structural and magnetic studies. *Polyhedron*. **64**, 255–261 (2013).

[24] Su, K. et al. Synthesis and characterization of decanuclear Ln(III) cluster of mixed calix[8]arene-phosphonate ligands (Ln = Pr, Nd). *Inorg. Chem. Commun*. **54**, 34–37 (2015).
[25] Hussain, F.; Patzke, G. R. Self-assembly of dilacunary building blocks into high-nuclear $[Ln_{16}As_{16}W_{164}O_{576}(OH)_8(H_2O)_{42}]^{80-}$ (Ln = Eu^{III}, Gd^{III}, Tb^{III}, Dy^{III}, and Ho^{III}) polyoxotungstates. *CrystEngComm.* **13**, 530–536 (2011).
[26] Li, Z. et al. Four-Shell Polyoxometalates Featuring High-Nuclearity Ln_{26} Clusters: Structural Transformations of Nanoclusters into Frameworks Triggered by Transition-Metal Ions. *Angew. Chem. Int. Ed*. **56**, 2664–2669 (2017).
[27] Zhao, L.; Xue, S.; Tang, J. A Dodecanuclear Dysprosium Wheel Assembled by Six Vertex-Sharing Dy_3 Triangles Exhibiting Slow Magnetic Relaxation. *Inorg. Chem.* **51**, 5994−5996 (2012).
[28] Miao, Y.-L. et al. Two novel Dy_8 and Dy_{11} clusters with cubane $[Dy_4(\mu_3\text{-}OH)_4]^{8+}$ units exhibiting slow magnetic relaxation behavior. *Dalton Trans.* 40, 10229–10236 (2011).
[29] Luo, X.-M. et al. Exploring the Performance Improvement of Magnetocaloric Effect Based Gd-Exclusive Cluster Gd_{60}. *J. Am. Chem. Soc.* **140**, 11219–11222 (2018).
[30] Dinca, A. S. et al. Aggregation of $[Ln^{III}{}_{12}]$ clusters by the dianion of 3-formylsalicylic acid. Synthesis, crystal structures, magnetic and luminescence properties. *Dalton Trans.* **48**, 1700–1708 (2019).
[31] Piquer, L. R. et al. Hysteresis enhancement on a hybrid Dy(III) single molecule magnet/iron oxide nanoparticle system. *Inorg. Chem. Front*. **6**, 705–714 (2019).
[32] Ma, X.-F. et al. Formation of nanocluster $\{Dy_{12}\}$ containing Dy-exclusive vertex-sharing $[Dy_4(\mu_3\text{-}OH)_4]$ cubanes via simultaneous multitemplate guided and step-by-step assembly. *Dalton Trans.* 48, 11338–11344 (2019).
[33] Li, X.-Y. et al. A Giant Dy_{76} Cluster: A Fused Bi-Nanopillar Structural Model for Lanthanide Clusters. *Angew. Chem. Int. Ed.* **58**, 10184–10188 (2019).
[34] Wang, K. et al. A single-stranded $\{Gd_{18}\}$ nanowheel with a symmetric polydentate diacylhydrazone ligand. *Chem. Commun.* **52**, 8297–8300 (2016).
[35] Wang, K. et al. Diacylhydrazone-assembled $\{Ln_{11}\}$ nanoclusters featuring a “double-boats conformation” topo-logy: synthesis, structures and magnetism. *Dalton Trans.* **47**, 2337–2343 (2018).
[36] Zhou, Y.-Y. et al. New Family of Octagonal-Prismatic Lanthanide Coordination Cages Assembled from Unique Ln_{17} Clusters and Simple Cliplike Dicarboxylate Ligands. *Inorg. Chem.* **55**, 2037−2047 (2016).
[37] Biswas, S. et al. Homometallic Dy^{III} Complexes of Varying Nuclearity from 2 to 21: Synthesis, Structure, and Magnetism. *Chem. Eur. J.* **23**, 5154–5170 (2017).
[38] Westin, L. G.; Kritikos, M.; Caneschi, A. Self assembly, structure and properties of the decanuclear lanthanide ring complex, $Dy_{10}(OC_2H_4OCH_3)_{30}$. *Chem. Commun.* 1012–1013 (2003).
[39] Ke, H. et al. A Dy_{10} Cluster Incorporates Two Sets of Vertex-Sharing Dy_3 Triangles. *Chem. Eur. J.* **15**, 10335–10338 (2009).
[40] Miao, Y.-L. et al. Chloride templated formation of $\{Dy_{12}(OH)_{16}\}^{20+}$ cluster core incorporating 1,10-phenanthroline-2,9-dicarboxylate. *CrystEngComm.* **13**, 3345–3348 (2011).
[41] Tian, H.; Bao, S.-S.; Zheng, L.-M. Cyclic single-molecule magnets: from the odd-numbered heptanuclear to a dimer of heptanuclear dysprosium clusters. *Chem. Commun.* **52**, 2314–2317 (2016).
[42] Thielemann, D. T. et al. Peptoid-Ligated Pentadecanuclear Yttrium and Dysprosium Hydroxy Clusters. *Chem. Eur. J.* **21**, 2813–2820 (2015).
[43] Richardson, P. et al. $[Ln_{16}]$ complexes (Ln = Gd^{III}, Dy^{III}): molecular analogues of natural minerals

such as hydrotalcite. *Dalton Trans.* **47**, 12847–12851 (2018).
[44] Lin, W.-Q. et al. Lanthanide Oxide Clusters: From Tetrahedral $[Dy_4(\mu_4\text{-}O)]^{10+}$ to Supertetrahedral $[Ln_{20}(\mu_4\text{-}O)_{11}]^{38+}$ (Ln = Tb, Dy, Ho, Er). *Chem. Eur. J.* **19**, 12254–12258 (2013).
[45] Tian, H. et al. Reversible ON–OFF switching of single-molecule-magnetism associated with single-crystal-to-single-crystal structural transformation of a decanuclear dysprosium phosphonate. *Chem. Sci.* **9**, 6424–6433 (2018).
