# Supplementary material for: Assembly of Dy60 and Dy30 cage-shaped nanoclusters
Source: Commun Chem. 2020 Mar 6;3:30. doi: 10.1038/s42004-020-0276-3 (PMC9814749; doi:10.1038/s42004-020-0276-3)
Supplement: Supplementary file 2 — Description of Additional Supplementary Files [file 42004_2020_276_MOESM2_ESM.pdf]

Description of Additional Supplementary Files

File Name: Supplementary Data 1 Description: crystallographic information file for compound **Dy_{30}.**

File Name: Supplementary Data 2 Description: crystallographic information file for compound **Dy_{60}.**

File Name: Supplementary Data 3 Selected bond lengths (Å) and angles (°) of complexes **Dy_{30}** and **Dy_{60}.**
